# Advanced Materials-Based Nanofiltration Membranes for Efficient Removal of Organic Micropollutants in Water and Wastewater Treatment

**DOI:** 10.3390/membranes15080236

**Published:** 2025-08-05

**Authors:** Haochun Wei, Haibiao Nong, Li Chen, Shiyu Zhang

**Affiliations:** 1 Guangxi Key Laboratory of Chemistry and Engineering of Forest Products, School of Chemistry and Chemical Engineering, Guangxi Minzu University, Nanning 530006, China; weihaochun@stu.gxmzu.edu.cn (H.W.); nonghaibiao@stu.gxmzu.edu.cn (H.N.); 2 Department of Chemical and Biomolecular Engineering, National University of Singapore, Singapore 117585, Singapore

**Keywords:** organic micropollutants, nanofiltration, membrane materials, wastewater treatment, efficient separation

## Abstract

The increasing use of pharmaceutically active compounds (PhACs), endocrine-disrupting compounds (EDCs), and personal care products (PCPs) has led to the widespread presence of organic micropollutants (OMPs) in aquatic environments, posing a significant global challenge for environmental conservation. In recent years, advanced materials-based nanofiltration (NF) technologies have emerged as a promising solution for water and wastewater treatment. This review begins by examining the sources of OMPs, as well as the risk of OMPs. Subsequently, the key criteria of NF membranes for OMPs are discussed, with a focus on the roles of pore size, charge property, molecular interaction, and hydrophilicity in the separation performance. Against that background, this review summarizes and analyzes recent advancements in materials such as metal organic frameworks (MOFs), covalent organic frameworks (COFs), graphene oxide (GO), MXenes, hybrid materials, and environmentally friendly materials. It highlights the porous nature and structural diversity of organic framework materials, the advantage of inorganic layered materials in forming controllable nanochannels through stacking, the synergistic effects of hybrid materials, and the importance of green materials. Finally, the challenges related to the performance optimization, scalable fabrication, environmental sustainability, and complex separation of advanced materials-based membranes for OMP removal are discussed, along with future research directions and potential breakthroughs.

## 1. Introduction

Since the 20th century, with the rapid increase in global population and the continuous expansion of industry, the discharge of OMPs such as antibiotics, analgesics/antipyretics/anti-inflammatories, insecticides, antiepileptics, β-receptor blockers/agonists, preservatives, and hormones into the environment has been rising. The OMPs mainly include PhACs, EDCs, and PCPs, which pose potential hazards not only to human health but also to entire ecosystems [1,2]. As early as the 1960s–1970s, Stumm-Zollinger et al. first proposed the adverse effects of non-biodegradable OMPs in municipal wastewater on the environment. With the development of modern civilization, an increasing number of researchers, organizations, and governments have begun to focus on the sustainable development of human living environments [3]. For example, government and non-government institutions such as the World Health Organization (WHO), the United Nations Environment Programme (UNEP), the Food and Agriculture Organization of the United Nations (FAO), and the International Water Resources Association (IWRA) are taking proactive actions to establish legal frameworks aimed at improving and protecting the quality of freshwater resources. As a result, a large number of studies and funding packages on OMPs have emerged, covering but not limited to the following aspects: detection of OMPs in natural water areas [4], research on toxicity analytical methods for OMPs in water [5], investigation of accumulation patterns of OMPs in the environment [6,7], and research on OMP treatment technologies [8]. To remove OMPs from water, researchers have proposed various advanced technologies, including adsorption, advanced oxidation processes, the electrochemical/electro-oxidation method, and the ion exchange method [8,9,10,11,12,13]. However, these methods face significant challenges due to factors such as high energy consumption and large maintenance and operational costs.

Compared with traditional treatment technologies, NF is a pressure-driven membrane separation process that retains specific ions, particles, or molecules based on the pore size, charge property, molecular interaction, and hydrophilicity of membranes [14,15,16]. Consequently, NF membranes can be specifically designed and regulated according to different OMPs to achieve precise molecular separation. Due to these properties, the demand for NF membranes in various OMP separation applications is steadily increasing (Table 1). In recent years, reviews of research on OMPs have mainly focused on their hazards and treatment technologies [3,17,18,19,20]. Although some literature has reviewed the application progress of membrane technologies in the removal of emerging OMPs [17,18], to the best of the authors’ knowledge, no reviews have specifically focused on the research progress of emerging NF membrane materials for OMP separation. Therefore, this article discusses (1) the sources, risks, and treatment technologies of OMPs; (2) key technical properties of NF membranes; (3) emerging membrane materials for OMP NF over the past 15 years; (4) the unique physicochemical properties of membrane materials, their regulation strategies, and corresponding OMP separation performance and mechanisms; and (5) a summary of issues in the application of emerging materials for OMP NF, along with prospects for future development.

## 2. OMPs in Water and Wastewater

In this review, the OMPs studied primarily encompass PhACs and EDCs from the chemical and pharmaceutical industries and PCPs from household chemical industries. We present a complete list of systematically categorized OMPs that appear in this review in Table 1. Original and review articles were identified using scientific search engines including Web of Science, Scifinder, and Google Scholar.

### 2.1. The Source of OMPs

**Figure 1:**
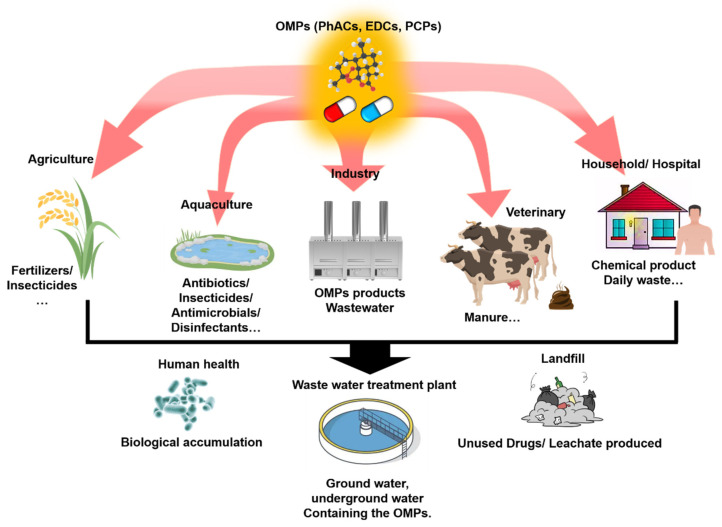
Schematic diagram of the sources of OMPs.

PhACs and EDCs as environmental contaminants encompass a broad spectrum of substances, including human and veterinary drugs (e.g., antibiotics), analgesics, insecticides, stimulants, and antiepileptics. In particular, EDCs are a collection of chemicals impacting sexual development, reproduction, and endocrine systems of wildlife and humans even at extremely low concentrations [19,20]. Currently, approximately 3000 compounds are utilized as pharmaceuticals, with annual global production exceeding hundreds of thousands of metric tons [21,22]. The worldwide pharmaceutical revenue surged from USD $390.2 billion in 2001 to a projected USD $1135 billion by 2025, reflecting a near threefold increase in drug consumption over the past 15 years [23]. The largest source of PhACs and EDCs are the production processes of the chemical and pharmaceutical industries. Water serves as a critical resource in chemical and pharmaceutical manufacturing, essential for production, material processing, and cooling operations. In the pharmaceutical sector, the E-factor (the ratio of the total mass of waste generated to the mass of the target product) ranges from 50 to 100 kg/kg [6]. Those in the chemical and pharmaceutical industries are significantly higher than that of the petrochemical industry (Table 2). This disparity arises from the inherent molecular complexity of PhACs and EDCs and the extensive chemical transformations required for their synthesis. As a result, the pharmaceutical industry generates large quantities of water containing PhACs and EDCs, encompassing potable water, process water, utility feed water, recycled water, wastewater, by-product treatment water, odor control water, desalinated water, and irrigation water. Notably, an estimated 50% of pharmaceutical wastewater worldwide is discharged without specialized treatment. In addition, PhACs and EDCs can enter aquatic ecosystems through multiple pathways: septic system failures, sewer line leaks, both permitted and accidental discharges, illegal dumping activities, cross-connections between sanitary and storm sewers, and improper management of pet and livestock waste (Figure 1). These contamination routes contribute to the widespread presence of pharmaceutical residues in surface waters, groundwater systems, and aquifers, raising significant environmental and public health concerns [24]. In regard to the detection of PhACs and EDCs in various environmental matrices—including sewage, surface water, groundwater, and drinking water—their concentrations typically remain in the parts-per-trillion (ppt) to parts-per-billion (ppb) range [24]. Although these compounds are present at trace levels, prolonged exposure may exert harmful effects on human health, aquatic ecosystems, and plant life.

PCPs, encompassing a broad spectrum of chemical formulations including disinfectants, perfumes, household chemical agents, emollients, hair care products, surfactants, food adjuvants, cosmetics, and sunscreens, are extensively utilized to augment the quality of daily human life [29]. The global cosmetics market, a significant subset of PCPs, was valued at over EUR 500 billion in 2018, with projections indicating a further escalation in value in the ensuing years [30]. In the context of daily human activities, the utilization of PCPs results in the introduction of these chemical compounds into wastewater treatment facilities in diverse, often complex mixtures. These mixtures are subsequently discharged into the environment via domestic sewage systems. Beyond the waste streams associated with consumer usage, the disposal of PCPs during the manufacturing processes of daily-use chemicals, medical protection, and food industry additives also contributes to the contamination of freshwater ecosystems, primarily due to the pervasive overuse of preservatives [30]. A substantial proportion of PCPs that are reintroduced into natural ecosystems exhibit non-biodegradable characteristics, rendering them refractory to conventional wastewater treatment methodologies, which typically encompass primary, secondary, and tertiary treatment stages. This resistance to degradation leads to the persistent accumulation of these chemicals in aquatic environments, with potential deleterious ecological consequences.

### 2.2. The Risk of OMPs

The original purpose of OMPs was to enhance people’s living standards. However, when these compounds appear in inappropriate environments, they may pose potential risks to healthy populations or individuals not intended to use these substances.

PhACs have been detected in various environmental matrices, including wastewater, surface water, groundwater, and drinking water, albeit at concentrations typically ranging from parts per trillion to parts per billion. Designed to be biologically active even at low doses, PhACs can induce a range of abnormalities in non-target species upon prolonged exposure. In aquatic environments, PhACs pose potential hazards to aquatic organisms and animals, while long-term ingestion of vegetables and meat contaminated with these compounds may adversely affect human health. For instance, mixtures of estradiol and 4-nonylphenol have been shown to exert synergistic effects in juvenile rainbow trout, inducing vitellogenin production. Mixtures of carbamazepine and fibrates have demonstrated far greater toxicity to Daphnia species than individual compounds at the same concentrations [31]. Prolonged exposure to anesthetics may impair the body’s ability to metabolize substrates, resulting in disproportionately elevated plasma concentrations and consequent toxicity [32]. β-blockers inhibit the interaction between catecholamines and β-adrenergic receptors (G-protein-coupled receptors), reducing calcium influx into cardiomyocytes and thereby lowering blood pressure [33]. Furthermore, these agents may impede cell regeneration [34]. Complete degradation of ibuprofen typically leads to the formation of metabolites such as hydroxy-ibuprofen and carboxy-ibuprofen, which may exhibit toxicities comparable to or exceeding those of the parent compound [35].

EDCs are among the emerging compounds of concern, known for their effectiveness in treating human diseases and also for their ability to disrupt biosynthesis, transport, and metabolism mechanisms. Such disruptions can manifest as precocious puberty, infertility, obesity, diabetes, reproductive disorders, cancerous tumors, and immune system, hormone activity, and organ dysfunction. Humans, especially children and unborn infants, are at the highest risk of harm from EDCs. Certain pharmaceutical EDCs can disrupt growth and development in children, leading to hormonal imbalances, metabolic disorders, and even genetic mutations, which in severe cases can result in death [36]. Analysis of post-mortem hypothalamic brain tissue from 24 child cases revealed the presence of seven EDCs, including bisphenol A, triclosan, triclocarban, and methyl, ethyl, n-propyl, and benzyl parabens [37]. Therefore, the improper discharge and use of these compounds pose not only a risk to patient health but also a broader health threat to the general population.

PCPs are widely used chemical products aimed at maintaining personal hygiene and preventing the spread of infectious diseases. Among these, preservatives are particularly prevalent, such as parabens. These preservatives exhibit effective inhibitory activity against a wide range of bacteria and fungi, including Gram-positive and Gram-negative bacteria, molds, and yeasts, in daily-use products [38,39,40]. However, preservatives can also suppress the synthesis of DNA and RNA, inactivate bacterial enzymes, and disrupt the lipid layers of cell membranes, leading to membrane rupture [41,42]. Prior to the publication of Darbre et al., PCPs containing preservatives were generally regarded as safe, but the study suggested a potential association between PCP use and the accumulation of preservatives in breast cancer tissue [43].

### 2.3. Treatment Technologies for OMPs

Due to the cumulative effects of OMPs in the environment, various technologies have been developed to separate them from water and wastewater. Although conventional wastewater treatment processes have been widely applied in industry, these biologically based technologies have shown limited efficiency in removing OMPs [44]. Traditional wastewater treatment plants were not originally designed to completely remove OMPs. Thus, treated water often contains trace amounts of these pollutants [45]. This residual contamination can lead to bioaccumulation in animals and plants, ultimately posing risks to human health. Development of activated sludge processes can effectively remove certain OMPs through biodegradation and adsorption, particularly benefiting drugs with high adsorption coefficients, which readily separate with sludge and sediments [46].

In addition to traditional approaches for OMP removal, the past 15 years have witnessed the emergence of novel water treatment technologies aimed at addressing OMPs. Among newer OMP removal techniques, ion exchange and adsorbent separation have received considerable attention in research [47,48]. Ion-exchange resins capture OMPs in ionic form, but their porous structures can foster microbial growth, creating challenges in regenerating these resins and adsorbents. Chlorination effectively degrades OMPs through reactions between residual chlorine and highly reactive functional groups; however, chlorination by-products themselves can be biologically toxic. Advanced oxidation processes (AOPs) have been extensively developed, with techniques such as the Fenton oxidation method and ozone/hydrogen peroxide oxidation demonstrating particularly high removal efficiencies [49]. In addition, biological treatment methods have also received increasing attention [50]. Notably, NF technologies have attracted significant attention due to their superior separation performance, alignment with green and environmentally friendly principles, and favorable economic feasibility [51,52]. Membrane separation technologies, including NF, reverse osmosis (RO), and forward osmosis (FO), can overcome traditional treatment limitations regarding the removal of OMPs. Additional advantages include compact system designs, modularity, ease of scalability, lower chemical usage, and consistent performance. By being used in conjunction with other physical, chemical, or biological processes, NF can target specific treatment goals, thereby improving overall efficiency while minimizing energy input and membrane fouling. Therefore, nanofiltration can serve as a valuable complementary technology within multi-barrier or hybrid treatment systems.

## 3. Key Properties of NF Membranes for Separation

Based on their compositional structures, NF membranes can be broadly categorized into integrally skinned asymmetric (ISA) membranes prepared via phase inversion, thin-film composite (TFC) membranes, and thin-film nanocomposite (TFN) membranes, among which TFC membranes are gradually becoming the mainstream in commercially available NF membranes [53,54]. TFC and TFN membranes exhibit an asymmetric structure, with the entire membrane consisting of a thin selective layer on the surface and a porous support membrane at the bottom. In NF membranes, the primary separation function is predominantly carried out by the thin selective layer on the surface. Consequently, the structure and properties of the NF selective layer often play a decisive role in the overall performance of the NF membrane [55,56]. Designing and regulating the structure of the NF selective layer is currently the principal direction for preparing high-performance NF membranes.

**Figure 2:**
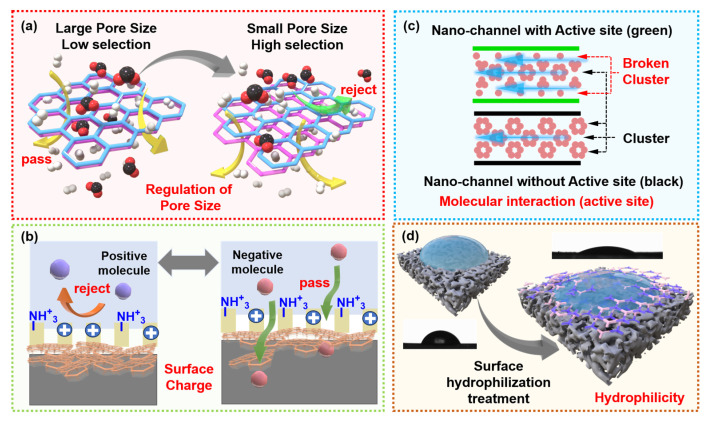
Schematic diagram of key properties of NF membranes.

The separation mechanisms of NF membranes for the rejection of target molecules primarily encompass size sieving, Donnan exclusion, and molecular interactions. Consequently, NF membranes must possess the following properties: appropriate pore size for sieving, corresponding surface charge, functional group interaction sites, and a hydrophilic surface [57]. For NF membranes intended for industrial applications, stability is also of paramount importance. The performance of a membrane is dictated by its structural and physicochemical properties including pore size, surface charge, active site, and hydrophilicity (Figure 2). The following section will analyze how to achieve efficient removal of OMPs by modifying these properties of NF membranes.

### 3.1. Pore Size

**Figure 3:**
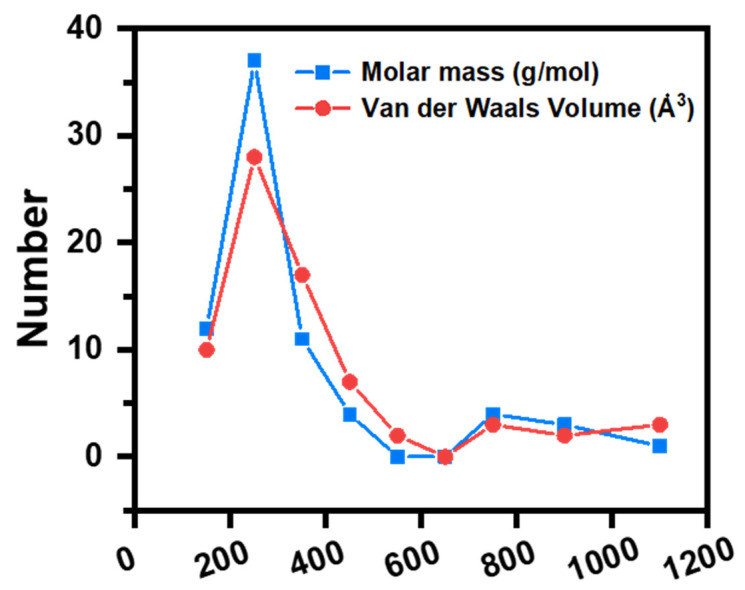
Distribution of OMPs’ quantity for NF (based on Van der Waals volume and molar mass).

As shown in Figure 3, over the past 15 years, the molecular weight of OMPs separated using NF membranes in the literature has predominantly ranged from 200 to 350 g/mol, with their van der Waals volumes concentrated between 200 and 400 Å^3^. Therefore, selecting NF membranes with an appropriate pore size distribution is crucial for the separation of OMPs.

For the ISA membranes, the preparation of NF membranes via the phase inversion method offers notable advantages, including a straightforward operational procedure and facile scalability for large-scale production. The final pore structure of the polymer film is determined by the combined effect of the thermodynamic properties of the casting liquid and the kinetic properties of the phase transformation process. The parameters that can affect the above two major properties mainly include the polymer concentration [58], types of solvents and additives [59,60], additive content [61], and some process parameters [62,63]. Among them, adding appropriate additives to the casting solution is a very effective and convenient means to regulate the structure and performance of polymer membranes. The commonly used additives for preparing polymer membranes can be classified into the following three categories: organic additives, inorganic additives, and non-solvent additives. In recent years, the preparation of NF membranes via the non-solvent-induced phase separation (NIPS) method using porous materials as membrane materials has emerged as a research focus, as it enables the modulation of separation membrane structures to obtain ISA membranes with finer pore structures [64].

Unlike the structure of ISA membranes, TFC membranes are mainly composed of a thin selection layer formed by one polymer and a support membrane made of another polymer material. The selection layer and the support membrane have different chemical structures. Since the selection layer and support layer in the TFC membrane can be made of different materials, compared with the ISA membrane, the range of available membrane materials is greatly increased. Moreover, compared with ISA membranes, the thin-layer structure of the selective layer of TFC membranes can be regulated independently, greatly expanding the technical means of membrane structural regulation. At present, the preparation methods of TFC mainly include two categories: the coating method and interfacial polymerization (IP) method [65]. Since the vast majority of polyamide selective layers are prepared by IP methods, the IP process plays a decisive role in the pore size of TFC membranes [66]. Its regulation strategies encompass the (1) adjustment of IP kinetics; (2) adjustment of monomer types; and (3) adjustment of IP operation methods.

For the TFN membranes, after nanoparticles (NPs) with special pore structures and functions are entered into the polymer selection layer, new separation channels can be generated in the polymer selection layer or the structure of the polymer selection layer can be changed [56]. In 2013, Sorribas et al. first introduced MOFs as fillers in TFN membranes [67]. In this study, MOF NPs [ZIF-8, MIL-53(Al), NH_2_-MIL-53(Al), and MIL-101(Cr)] with diameters ranging from 50 to 150 nm were embedded into the polyamide (PA) layer through IP. The results demonstrated that as the porosity and pore size of the MOFs increased, solvent permeability also increased, indicating that the pores in the MOFs provided preferential pathways for solvent transport.

### 3.2. Surface Charge

As shown in Table 3, over the past 15 years, the literature reports the charge characteristics of OMPs separated using NF membranes. The fabrication of charged membranes represents an effective strategy for enhancing membrane separation performance [68]. It is necessary to select NF membranes with appropriate surface charges corresponding to the charge characteristics of OMPs, utilizing electrostatic repulsion to achieve optimal OMP rejection [69,70,71]. Based on the presence or absence of electrical charges on the membrane surface, NF membranes can be classified into charged membranes and neutral membranes. In addition to possessing the physical sieving functionality inherent to neutral membranes, charged membranes exhibit unique electrostatic adsorption and repulsion effects. These distinctive properties enable the separation of smaller-sized substances using membranes with larger pore sizes and facilitate the separation of components with similar molecular weights but differing charge characteristics. Furthermore, the incorporation of charged groups into the membrane matrix enhances its hydrophilicity. Concurrently, the charge repulsion effect among like charges amplifies the membrane’s antifouling resistance. Consequently, charged membranes confer distinct advantages over neutral membranes in terms of permeability, antifouling capability, and selective permeability [72].

The preparation methodologies for positively/negatively charged membranes can be systematically categorized into pre-modification approaches and post-modification approaches based on the chronological sequence of the fabrication process. The pre-modification strategies encompass IP of cationic/anionic monomers, layer-by-layer (LbL) assembly, and co-blending techniques [73,74]. IP is a prevalent method for membrane fabrication, and employing positively charged monomers in this process serves as an effective strategy for preparing positively charged membranes. Polyethyleneimine (PEI) is one of the most extensively utilized positively charged modifying agents. Chen et al. endowed the membrane with charge properties by introducing PEI during the IP process, thereby achieving a superior NF separation performance [75]. The LbL assembly technique represents an effective approach for the synthesis of positively charged membranes. Typically, it involves the alternate deposition of cationic and anionic polyelectrolyte solutions onto a charged substrate, thereby enabling relatively precise control over the membrane’s structure and composition [76]. Yang et al. alternately assembled PEI and PAA to form a thin and dense PEI/PAA layer on the polyvinylidene fluoride (PVDF) substrate, achieving a retention rate of 97.9% for the positively charged dye [77]. For co-blending techniques, Wang et al. pioneered the synthesis of polycationic liquids and subsequently blended them with PVDF to fabricate a positively charged membrane. The resultant membrane demonstrated exceptional separation efficiency, achieving a retention rate of 99.4% for oil droplets and 85.6% for bovine serum albumin (BSA) [78].

Conversely, post-modification strategies encompass surface functionalization via coating, crosslinking-induced network formation, and radical-mediated grafting techniques [79,80,81]. These methodologies are applied to pre-synthesized membrane substrates to confer charge density on the membrane surface or within its porous matrix, thereby tailoring its separation performance. The surface coating method involves coating a solution containing high-molecular-weight polymers and active monomers onto the surface of a support through immersion, filtration, or casting and then fixing it on the surface of the substrates by ultraviolet or heat treatment to prepare the membranes [82,83]. Zhao et al. modified PEI with gallic acid (GA) and then coated the modified GA-PEI solution on the hydrolyzed PAN ultrafiltration membrane. The prepared positively charged NF membrane achieved retention rates of 97.3% for methyl blue and 97.1% for Congo red, which was beneficial for spinning the treatment and reuse of wastewater [84]. The crosslinking method refers to a technique in which polymer chains are linked together through chemical bonds under the influence of light, heat, radiation, and crosslinking agents, forming a network or solid structure. Modifying polymers through crosslinking can enhance their chemical resistance and mechanical strength. Chen et al. achieved the incorporation of fluorinated functional groups by crosslinking the COFs with a PA layer. The resultant TFNi-C1(2.1)-0.9 membrane demonstrated a removal efficiency exceeding 99.2% for PhACs while maintaining satisfactory flux [85]. Chemical grafting is a method that generates viable sites on the membrane surface through physical or chemical treatment and introduces positively charged monomers for grafting polymerization, thereby forming a grafted layer with separation properties [86].

### 3.3. Molecular Interaction

In NF membranes, the introduction of chemically active sites (such as catalytic degradation sites and chemical adsorption sites) or functional groups capable of specific interactions with target molecules (such as π-π interactions and coordination interactions) within the pores endows the membrane with separation mechanisms that go beyond traditional electrostatic attraction/repulsion, exhibiting unique molecular interaction characteristics. Certain porous materials, such as MOFs and COFs, are particularly well-suited for the precise modulation of active sites. Their highly ordered and tunable porous structures, combined with a large specific surface area, provide an abundance of chemically active binding sites [87,88]. Strategies for introducing active adsorption or reaction sites include 1. Utilizing organic ligands or metal ions/clusters containing active sites to directly incorporate them into the internal structure of the porous base membrane during the preparation of the organic framework. Ideally, this can achieve complete modification of active sites. However, the increased presence of active sites leads to greater steric hindrance for reactions, which raises the difficulty of preparing such porous materials. 2. Introducing molecules or molecular chains with active sites onto the surface of porous materials via chemical grafting methods, such as click chemistry reactions. However, the grafting efficiency of such chemical grafting methods is usually low, typically around 5%, resulting in an overall insufficient activity of the porous material. 3. Physically blending/coating active site-containing compounds with the porous base membrane. This method is simple and effective. However, due to the lack of chemical bonding, there is a risk that the active site-containing compounds may detach from the porous base membrane, reducing the membrane’s regeneration performance and long-term stability. NF membranes with special molecular interactions are primarily applied in the fields of catalytic degradation-based membrane separation and chemical adsorption-based membrane separation. For example, Li et al. introduced Pd nanoclusters into covalent organic cages, achieving dual functionality through the combination of molecular separation and catalytic activity [89]. This type of membrane facilitates precise molecular separation while enabling surface self-cleaning by leveraging the high catalytic activity of Pd nanoclusters (~0.7 nm), which react with any adsorbed contaminants to restore the membrane’s original performance. By incorporating various photocatalytic particles into the membrane, the issue of membrane fouling can be mitigated. Zhang et al. reviewed several design and modification strategies for enhancing the efficiency of photocatalysts, as well as different structural configurations of photocatalytic membrane systems [90].

### 3.4. Hydrophilicity

Hydrophilicity is crucial for the performance of water-based separation membranes. A membrane surface with good hydrophilicity can form strong interactions with water molecules, such as through hydrogen bonding and van der Waals forces, which promote water permeation and reduce the adsorption of contaminants. Computational studies by Zhang et al. demonstrated that preferential interactions between water and the membrane surface enable COF membranes with hydrophilic functional groups to exhibit higher water flux in NF applications [91]. In general, two key factors influencing the water flux of hydrophilic NF membranes are solid surface energy and surface roughness. Specifically, interfaces with lower solid surface energy tend to exhibit hydrophobic properties. For example, the introduction of fluorine-containing functional groups can significantly reduce surface energy, causing NPs to display a superhydrophobic interface state [92]. Conversely, researchers often employ hydrophilic modification by introducing functional molecules with –OH, –COOH, or –NH_2_ groups to the solid surface. For instance, Chen et al. utilized a click chemistry reaction to introduce cysteine, a molecule containing hydrophilic dual functional groups –NH_2_ and –COOH, into the active sites of COF, thereby imparting enhanced hydrophilicity to the TFN NF membrane and increasing pure water flux [75]. Surface roughness also plays an important role in enhancing hydrophilicity and water flux. An increase in the specific surface area of the membrane enhances the contact between the membrane surface and water molecules, thereby improving water permeation. For example, Chen et al. employed COF@PEI particles with a uniform morphology to modulate the microstructure of the PA layer during the interface polymerization (IP) process, which improved the membrane surface hydrophilicity and enhanced solvent flux [85]. It is worth mentioning that good hydrophilicity not only improves membrane flux but also significantly reduces the adhesion of foulants (such as organic matter, proteins, and microorganisms) on the membrane surface, thereby mitigating membrane fouling and extending membrane lifespan.

## 4. NF Membrane with Advanced Materials for Removal of OMs

**Figure 4:**
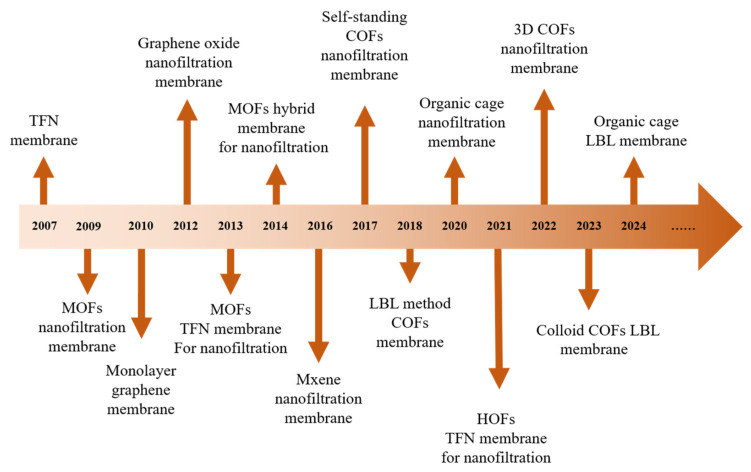
Research progress on NF membranes fabricated from advanced materials [67,93,94,95,96,97,98,99,100,101,102,103,106].

From the previous summary, it can be seen that certain intrinsic properties of membranes govern the transport mechanisms within or across membrane channels. These properties play a critical role in determining the transport rates of organic pollutants and ultimately influence the selective separation performance of the membrane. With the continuous advancement of membrane separation technologies, a growing variety of novel materials have been incorporated into NF membrane fabrication to achieve pore structures and properties better suited to specific separation scenarios (Figure 4) [93,94,95,96,97,98,99,100,101,102,103,104,105]. The introduction of these materials endows the membrane pores with unique structures and properties, thereby enabling more efficient separation of organic pollutants. This part provides a classified discussion of novel materials applied in the separation of organic pollutants over the past 15 years (2010–2025), including organic polymer (such as MOFs, COFs, and hydrogen-bonded organic framework (HOFs)), inorganic layered materials (such as graphene/graphene oxide, molybdenum disulfide, and MXene), as well as other emerging materials. We will introduce the categories and characteristics of these materials, with a particular focus on their membrane fabrication methods and OMP separation performance and mechanism. Finally, the development trends of each type of novel material will be analyzed, aiming to provide theoretical insights and practical guidance for the advancement of NF membranes based on emerging materials.

The data were obtained from Web of Science, Scifinder, and Google Scholar using the keywords “XX (materials name) AND nanofiltration AND (pharmaceutical OR personal care products OR endocrine disrupting compounds)” to retrieve relevant research articles between 2010 and 2025, with further manual screening to differentiate the techniques used (Date: May 2025).

### 4.1. Organic Framework

Organic frameworks represent a class of hybrid porous materials that are constructed by polytopic organic linkers through chemical bonds. As demonstrated in Table 4, based on the connectivity patterns of nodes and chemical bonds, these organic polymer materials can be broadly categorized into MOFs, COFs, porous organic frameworks (POFs), and HOFs. Organic frameworks possess long-range ordered porous channel structures; therefore, the degree of crystallinity is commonly used as an indicator of the structural order within their pore. Organic frameworks materials, particularly MOFs and COFs, hold significant application potential in fields such as adsorption, conduction, and membrane separation [104,105,107].

#### 4.1.1. MOFs

The synthesis of MOFs is typically carried out under mild conditions, involving the coordination of metal ions with organic ligands to form frameworks with specific architectures. This allows researchers to utilize reticular synthesis strategies, enabling the pre-synthetic design of ligands, nodes, and reaction conditions to tune the structure and properties of MOFs without altering their connectivity or topology. Notably, MOFs can be modified at the metal nodes or the organic linker after synthesis [108]. Accordingly, the pore structure of MOFs can be precisely engineered to meet the specific requirements of various separation environments (charge environments, pH environments, etc.).

**Figure 5:**
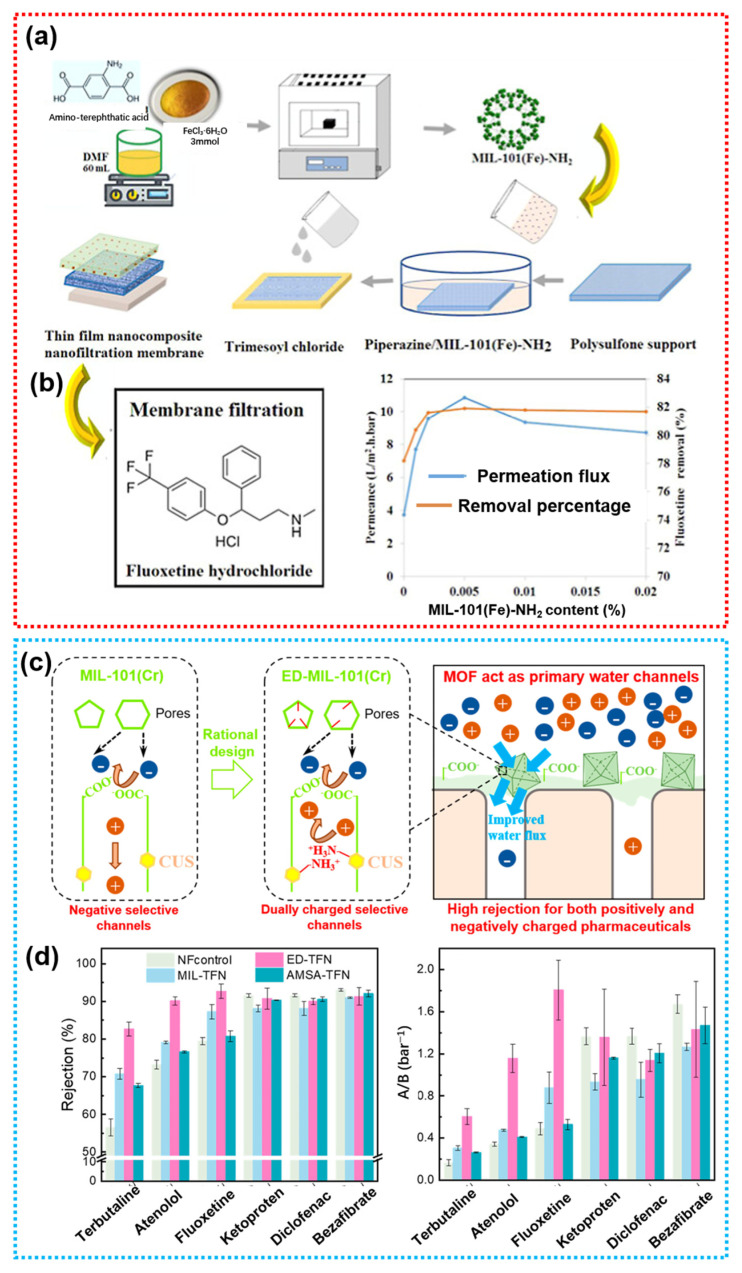
(**a**) Schematic illustration of the fabrication of an MIL-101(Fe)-NH_2_ TFN NF membrane. (**b**) Separation performance of fluoxetine hydrochloride using TFN membranes with different loading MIL-101(Fe)-NH_2_ [109], Copyright 2025, American Chemical Society. (**c**) Schematic of a dually charged MOF thin-film nanocomposite NF membrane for PhAC removal by rational design of MOF nanofiller. (**d**) Separation performance of PhACs using TFN membranes loaded with different MOFs [111], Copyright 2020, American Chemical Society.

In MOF-based TFN membranes, functional MOFs can be pre-dispersed in the aqueous phase and subsequently integrated into the polyamide (PA) layer via the IP process. This approach is straightforward, exhibits good reproducibility, and ensures a reliable performance. The intrinsic functional groups within the MOF pores can provide selective interactions with pharmaceutical molecules, enabling targeted rejection. For instance, Leila Roshanfekr et al. compared TFN membranes prepared using MIL-101(Fe)-NH_2_ with a positively charged amino-functionalized group to those using non-functionalized MOFs (MIL-101(Fe)) (Figure 5a,b) [109]. The study revealed that introducing a low concentration of charged MOFs into the aqueous phase significantly enhanced the membrane’s performance in removing antidepressants from pharmaceutical wastewater. Under optimal conditions, the water flux reached 9.16 LMH/bar, and the rejection rates for sertraline hydrochloride, paroxetine hydrochloride, and nortriptyline hydrochloride antidepressants were as high as 75.8%, 73.1% and 71.5%. For the negatively charged non-steroidal anti-inflammatory drugs (NSAIDs), Sara et al. reported that negatively charged MOF-808 with -COOH incorporated into TFN membranes via the IP process exhibited a superior rejection performance, compared to pristine TFC membranes without MOF-808 loading [110]. Specifically, the PES/MOF-808@PA TFN membrane achieved rejection rates of 88.76%, 84.51%, and 70.56% for aspirin, naproxen, and mefenamic acid, respectively, with a pure water flux of 6.04 LMH/bar. Dai et al. have grafted ethylenediamine (ED) onto the coordinatively unsaturated metal sites within MIL-101(Cr) (Figure 5c) [111]. The resulting ED-MIL-101(Cr) features strongly positively charged -NH_2_ within its pores and negatively charged -COOH on its surface. Based on this, a novel TFN membrane was rationally designed, incorporating a dual-charged MOF to effectively remove both positively and negatively charged PhACs. The dual-charge nature of the MOF NPs enabled the ED-MIL-101(Cr)-based TFN membrane to achieve high rejection (mostly above 90%) for both cationic PhACs (such as terbutaline, atenolol, and fluoxetine) and anionic PhACs (such as ketoprofen, diclofenac, and bezafibrate). Meanwhile, the water permeability of the ED-MIL-101(Cr) TFN membrane increased by 140% compared to the control membrane without MOF (Figure 5d). For the certain pH-sensitive drugs, Hajheidari et al. developed a novel approach by inducing pH responsiveness in both the membrane surface and sublayer. This was achieved by incorporating metal oxides with a closed-gate (CG) mechanism, such as copper oxide (Cu-pH-Res) and silica oxide (Si-pH-Res), or MOFs with an open-gate (OG) mechanism, such as ZIF-8 (Zn-pH-Res) and ZIF-67 (Co-pH-Res), into the two respective membrane layers. As a result, composite membranes SurCu^3^/SubSi_3_ and SurZn^3^/SubCo_3_ were synthesized. Under varying pH separation conditions, the metal centers in the MOFs interact with H^+^ ions from the feed solution within the membrane matrix, leading to a nearly fivefold enhancement in permeability under acidic conditions relative to a neutral pH [112].

**Figure 6:**
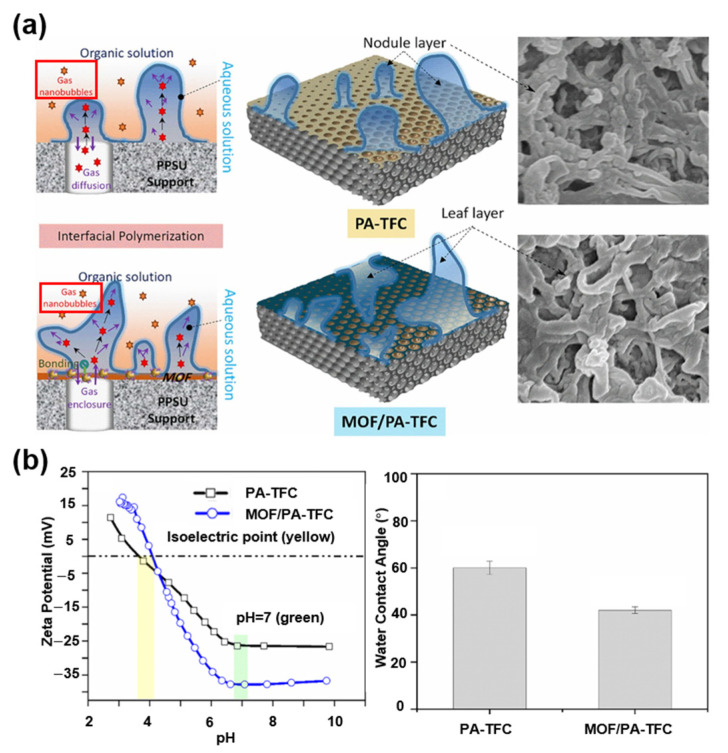
(**a**) Schematic illustration of the effects of incorporating MOF nanofillers on IP process and PA structure. (**b**) Surface charge and hydrophilicity of MOF/PA-TFC and control membranes [113]. Copyright 2020, Springer.

MOFs can influence the transport behavior of OMPs during the separation. In addition, some articles have focused on investigating the impact of MOFs on the IP process, aiming to tailor the structure and hydrophilic/hydrophobic properties of the selective layer to enhance the separation performance. For example, Shukla et al. developed a MOF/PA-TFC NF membrane for the removal of paracetamol, ibuprofen, and amoxicillin from simulated wastewater [113]. The author emphasizes the influence of MOF NPs on the hydrophilicity and membrane surface roughness (Figure 6). The improvement in membrane surface hydrophilicity was attributed to intermolecular hydrogen bonding between the surface functional groups of the MOFs and water molecules. On the other hand, the bonding effect generated by the nanoporous MOF layer on the surface could prevent the escape of degassed nanobubbles, leading to an increase in the effective filtration area of the PA membrane. As a result, the water transport rate through the polyamide layer was enhanced, thereby improving the overall water flux. Compared to conventional PA-TFC membranes, the newly developed membrane exhibited a high water flux of 3.5 LMH/bar along with a significantly enhanced rejection performance. The rejection rates for paracetamol, ibuprofen, and amoxicillin were 93%, 98%, and 99%, respectively. Zhao et al. fabricated TFN NF membranes via IP using three water-stable MOFs: MIL-53(Al), NH_2_-UiO-66, and ZIF-8. Membrane characterization revealed that the incorporation of MOFs reduced the degree of crosslinking and increased the membrane thickness and the roughness of the polyamide active layer. The results showed that the TFN NH_2_-UiO-66-BL-0.10% membrane achieved the highest water flux of 7.2 LMH/bar, approximately 1.3 times that of the control membrane.

#### 4.1.2. COFs

COFs are a class of porous crystalline materials constructed from organic molecules linked by covalent bonds. Both MOFs and COFs can be tailored to interact with specific OMPs; however, COFs allow for more straightforward chemical modification due to their fully organic composition. To target OMPs with different charges, COFs with appropriately functional groups can be selected to impart a suitable environment to the NF membrane, thereby establishing strong electrostatic interaction and significantly enhancing the separation performance of NF membranes. In addition, the pore sizes of COFs are generally better suited for the separation of OMPs, particularly those with higher molecular weights. For example, Banjerdteerakul et al. synthesized uniformly TpPa-SO_3_H nanosheets (negatively charged) using 2,5-diaminobenzenesulfonic acid [114]. Through a vacuum-assisted self-assembly method, COF nanosheets were stacked onto an yttria-stabilized zirconia hollow fiber to prepare a COF TpPa-SO_3_H composite membrane. The membrane exhibited excellent rejection of four negatively charged pharmaceutical pollutants: diclofenac (96.4%), ketoprofen (75.8%), naproxen (75.2%), and ibuprofen (79.4%). In contrast, the positively charged sulfamethoxazole showed a relatively low rejection (57.4%). The presence of -SO_3_H in the COF suggests that electrostatic interactions are one of the dominant separation mechanisms in the composite membrane. Meanwhile, Kong et al. discovered that the prepared COF-LZU1 exhibits a negative charge, and as a result, the rejection rates of four drugs by COF-LZU1 vary with their molecular charge properties. Due to tetracycline’s high molecular weight and negative charge, it demonstrates the highest rejection rate. In contrast, propranolol, which carries a positive charge, shows the lowest rejection rate. The order of rejection rates is as follows: tetracycline > sulfadiazine > carbamazepine > propranolol. When the molecular weights are approximately the same, electrostatic repulsion causes negatively charged sulfadiazine to have a higher rejection rate compared to neutral carbamazepine and positively charged propranolol. Furthermore, by leveraging the porous nature of COFs, Liu et al. designed functional groups on TPB-DMTP COFs as active binding sites for the efficient capture of EDCs, cleverly exploiting the adsorption capability of COFs toward EDCs [115]. COFs were incorporated into the PA layer to prepare a novel TFN membrane with adsorption capabilities. After separation, the TFN-COF membrane can be rapidly and efficiently regenerated by washing with ethanol for a few minutes. Additionally, the porous structure of the COF nanofillers provides extra water channels, potentially overcoming the permeability–selectivity trade-off of NF membranes. The optimized TFN-COF membrane achieved removal rates of 98.3%, 99.1%, and 99.3% for bisphenol A, bisphenol AF, and sodium 2-naphthalenesulfonate, respectively, which are significantly higher than those of the original NF membrane (82.4%, 95.5%, and 96.4%, respectively).

**Figure 7:**
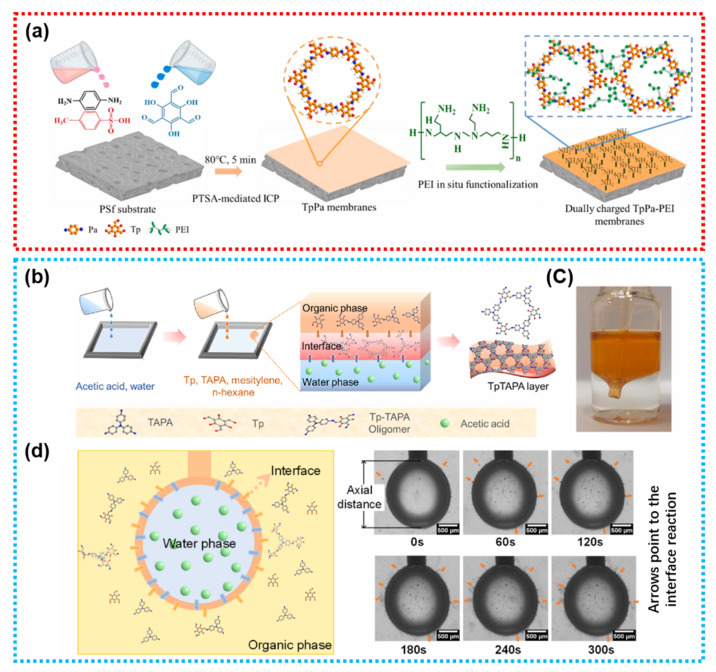
(**a**) Schematic diagram of the fabrication of PEI in situ TpPa membrane [116], Copyright 2025, Elsevier. (**b**) Schematic illustration of the fabrication of the TpTAPA/HPAN composite membrane. (**c**) Digital image of the self-standing TpTAPA thin film. (**d**) Schematic diagram of the OT-IP process on a pendant droplet [117], Copyright 2023, Elsevier.

Compared to the rigid framework of MOFs, COFs possess relatively flexible frameworks, which facilitate the fabrication of self-standing membranes with enhanced mechanical stability, as well as composite membranes with improved compatibility and higher loading capacity. Yue et al. utilized 1,3,5-triformylphloroglucinol (Tp) and p-phenylenediamine (Pa) to in situ crystallize a TpPa film on the surface of PSf substrate via a p-toluenesulfonic acid (PTSA)-mediated interfacial catalytic polymerization (ICP) method, synthesizing a defect-free pure COF layer (Figure 7a) [116]. Subsequently, the membrane was post-functionalized with PEI to enhance hydrophilicity and adjust membrane charge, thereby strengthening drug rejection. Notably, Zhao et al. also demonstrated the ability to fabricate a self-standing COF film through oligomer-triggered interfacial polymerization (OT-IP) process without a substrate (Figure 7b) [117]. As illustrated in Figure 7c, this COF membrane exhibits excellent mechanical strength, offering a flexibility that rigid MOFs struggle to achieve. Figure 7d depicts the pre-mixing of Tp and TAPA in an organic phase to form Tp-TAPA oligomers, which react at the phase interface under the catalysis of acetic acid in the aqueous phase. As acetic acid molecules, reactive monomers, and oligomers diffuse towards the phase interface, a dense thin film forms on the organic phase side. This approach enables the preparation of a pure COF film as a selective layer for NF. Other examples include Banjerdteerakul et al. forming a pure COF selective layer through the deposition of TpPa-SO_3_H nanosheets, and Kong et al. fabricating a COF layer on the surface of a base membrane via interfacial growth under light irradiation [114,118]. In summary, as a novel material for NF membranes, COFs can not only be used to modify TFN membranes by loading COF NPs into the PA layer, but also form a pure COF film as selective layers for NF membranes through deposition and in situ IP methods.

#### 4.1.3. POFs and HOFs

Compared with MOFs and COFs, other polymer-based porous materials have been less explored in the fabrication of NF membranes for the removal of OMPs, yet they exhibit significant potential for future development. For instance, POFs typically do not require monomers with high symmetry or well-defined reactive sites, emphasizing instead structural diversity and synthetic flexibility. As a result, POFs generally possess lower crystallinity. Compared to COFs, POFs can be synthesized under milder conditions, which is advantageous for industrial-scale production. For example, Liu et al. employed an in situ IP method under ambient temperature and pressure to fabricate flexible and continuous BILP-101x membrane directly on substrates [119]. By precisely tuning the synthesis process, the microstructure and separation performance of the BILP-101x membrane could be modulated. The optimized membranes demonstrated exceptionally highly water flux (~255 LMH/bar) along with an excellent NF performance. The unique antibacterial properties of BILP-101x and long-term operational stability make the composite membranes competitive and practical to use for NF applications in harsh pharmaceutical wastewater environments. In contrast, HOFs are constructed via hydrogen bonding interactions between organic building blocks, typically self-assembled at relatively mild conditions. HOFs form more flexible, yet structurally less robust frameworks, but their solubility and potential for self-healing offer promising opportunities for membrane processability. Jiang employed a facile IP strategy to fabricate a TFN membrane based on HOFs, using a PA/Nano-PFC-1 active layer on a polymer substrate [95]. This approach enabled the rapid construction of the HOF-based membrane within a short preparation time. The resulting membrane exhibited an ultrahigh water flux of 546.09 LMH/bar and demonstrated excellent rejection toward molecules commonly used as probes in biomedical analysis. Accordingly, the membranes exhibited long-term operational stability.

### 4.2. Inorganic Laminar Materials

In NF membranes, commonly used inorganic lamellar materials include graphene oxide (GO), MXenes, and molybdenum disulfide (MoS_2_) [120,121,122]. These materials possess unique sheet-like structures and can be readily fabricated into large-sized nanosheets. They also exhibit tunable physicochemical properties; their edges and defect sites serve as abundant anchoring points for functional groups such as −OH, −COOH, −NH_2_, etc. [123,124]. The introduction of these functional groups optimizes the surface charge, hydrophilicity/hydrophobicity, and interfacial compatibility of the nanosheets. On the one hand, the stacking of GO, MXene, and MoS_2_ nanosheets can lead to the formation of laminated membrane structures with high mechanical strength. Transport channels are formed via slit-like gaps at the nanosheet edges, interlayer spacing, and in-plane defects [125]. On the other hand, owing to their excellent film-forming ability and high dispersibility, these nanosheets can be incorporated into PA selective layers via the IP process, thereby enhancing water permeability or increasing the transport resistance for organic micropollutants.

#### 4.2.1. GO

**Figure 8:**
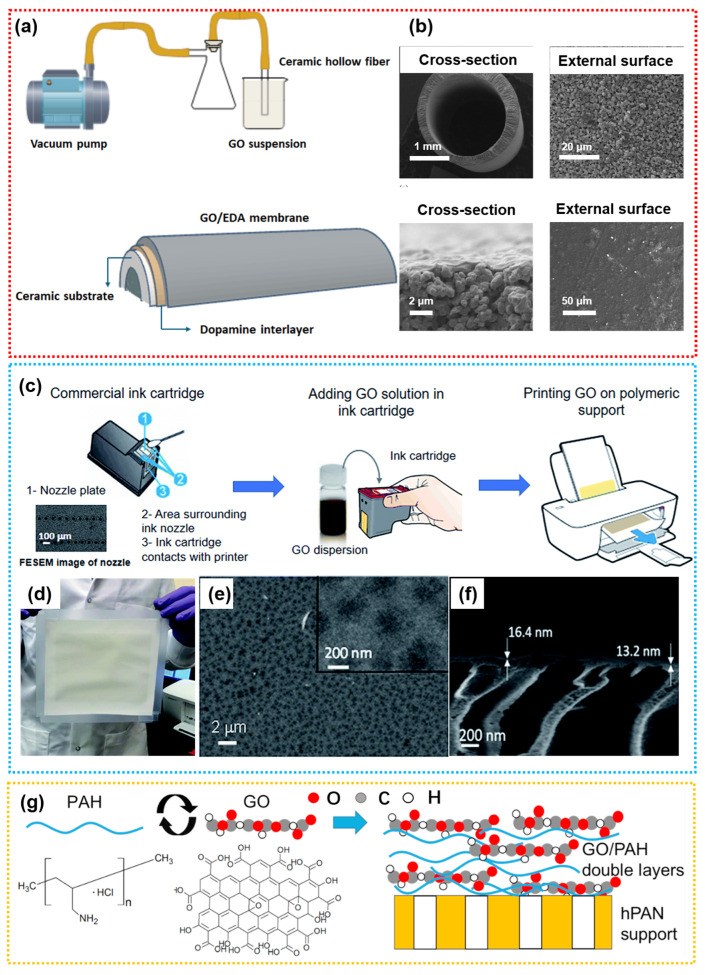
(**a**) Schematic diagram of the fabrication of GO/EDA membrane via vacuum-driven filtration [129], Copyright 2025, Elsevier. (**b**) SEM images of the ceramic hollow fibers and GO/EDA membrane. (**c**) Schematic diagram of the fabrication ultrathin GO membranes via inkjet printing. (**d**) Digital images of a printed GO membrane. SEM images showing surface (**e**) and cross-sectional (**f**) views of a GO membrane [130], Copyright 2017, Royal Society of Chemistry. (**g**) Schematic diagram of the layer-by-layer assembly process [131], Copyright 2017, Elsevier.

Different preparation methods of graphene oxide (GO) are associated with specific application characteristics. Currently, the main fabrication methods for GO-based composite membranes used in the separation of organic pollutants include vacuum/pressure-driven (V/P-D) filtration, coating, layer-by-layer self-assembly, and the IP process [18]. Among these, vacuum/pressure-driven filtration is the most popularly used technique for the large-scale preparation of GO-based lamellar membranes. This method offers several advantages, including a simple process, broad compatibility with various substrates, the ability to form lamellar membrane structures, controllable preparation conditions, high water flux, and excellent removal efficiency for OMPs. As such, they can serve as an advanced purification step for the separation of organic and inorganic salts from secondary effluents. Han et al. fabricated an NF membrane by depositing multiple layers of graphene onto a PES substrate via pressure-assisted deposition [126]. Their study on secondary effluents resulting from the chemical synthesis of pharmaceuticals in wastewater (including cephalosporin, anti-tumor drugs, and cardiovascular and cerebrovascular drugs) revealed that GO membranes exhibited high rejection rates for organic compounds (COD can be reduced from 176 to 42 mg O^2^/L) but relatively low rejection rates for inorganic salts. Consequently, GO membranes are particularly well-suited for the treatment of industrial wastewater containing high concentrations of mixed organic and inorganic substances, as they help prevent the mineralization and degradation of organics during the dehydration process. With the GO loading reduced to 14.4 mg/m^2^ (approximately 10 layers of GO), the cost of GO membranes becomes comparable to that of traditional polymer membranes. Chu et al. functionalized GO and modified commercial ceramic ultrafiltration membranes using a vacuum-assisted method [127]. Compared with the unmodified ceramic membranes, the GO-modified ceramic membranes exhibited enhanced hydrophilicity and a higher density of negative surface charges, resulting in improved rejection rates of ibuprofen and sulfamethoxazole. Hidalgo et al. prepared GO NF membranes via vacuum-assisted filtration, which were able to withstand a separation pressure of up to 20 bar during the separation of ibuprofen feed solution, indicating that the GO layer possesses excellent mechanical strength [128]. However, vacuum-assisted filtration on flat-sheet membranes is not suitable for the fabrication of large-area industrial membranes. To address this limitation, Cardoso et al. deposited GO onto alumina hollow fiber membranes (Figure 8a) [129]. The resulting alumina hollow fibers exhibited a desirable asymmetric pore structure, with a sponge-like outer layer. After forming a GO membrane with a thickness of (0.27 ± 0.02) μm, the average surface roughness of the ceramic hollow fiber decreased from (122.5 ± 5.6) nm to (42.32 ± 3.50) nm (Figure 8b). The membrane achieved a rifampicin rejection rate of (52.44 ± 7.01)%. This work provides a scalable example for the fabrication of GO NF membranes via vacuum or pressure-driven deposition methods. Compared to V/P-D deposition methods, techniques such as spray coating or 3D printing offer greater potential for industrial-scale production. Fathizadeh et al. employed inkjet printing to deposit ultrathin (7.5–60 nm), uniform GO NF membranes onto modified polyacrylonitrile (M-PAN) substrate, achieving membrane areas of up to 15 cm^2^ (Figure 8c–f) [130]. By adjusting the concentration of the GO “ink” and the printing time, the water flux and rejection performance of the printed GO membranes could be finely tuned. The printed membranes exhibited excellent separation performance in the removal of pharmaceutical pollutants (iodixanol, diclofenac sodium salt, 17α-ethynylestradiol, and gemfibrozil), with rejection rates of 95.2%, 83.0%, 80.1%, and 76.4%, respectively. The membranes also demonstrated outstanding stability, maintaining a 95% rejection rate for iodixanol over a long-term (120 h) operation test, with a water flux decline of less than 10%. GO membranes prepared via LBL self-assembly are more likely to form layered structures with alternating charges, enabling responsiveness to various separation environments, such as pH changes. As shown in Figure 8g, Oh et al. fabricated GO membranes via an LBL self-assembly method by alternately depositing positively charged poly(allylamine hydrochloride) (PAH, ~56,000 Da) and negatively charged GO nanosheets [131]. The key properties of the GO membranes, such as surface charge and interlayer spacing, varied with pH, resulting in distinct pH-dependent interfacial behaviors and separation mechanisms. These findings suggest that GO membranes prepared by this method can function as pH-responsive membranes.

It is worth noting that GO membranes fabricated via the vacuum/pressure-driven filtration method tend to detach from the substrate surface, exhibiting poor stability, and are difficult to backwash or reuse. In contrast, GO membranes prepared by the IP process demonstrate good stability and can be effectively backwashed and reused. Yadav et al. incorporated amine-functionalized GO into a 2% 3,5-diaminobenzoic acid (DABA) aqueous solution, providing essential functional groups to enhance surface interactions [132]. This facilitated proper crosslinking between –NH_2_ and acyl chlorides during the formation of the PA layer, thereby improving the structural stability of the overall NF membrane. Lin et al. used an m-phenylenediamine (MPD) solution containing 0.015 wt% GO as the aqueous phase to fabricate a TFC-GO membrane for the separation of six types of PPCPs [133]. Notably, the TFC-GO membrane exhibited resistance to hydrogen peroxide degradation while maintaining stable water flux, confirming that the IP method has a positive effect in preventing GO lamellar swelling and structural damage.

**Figure 9:**
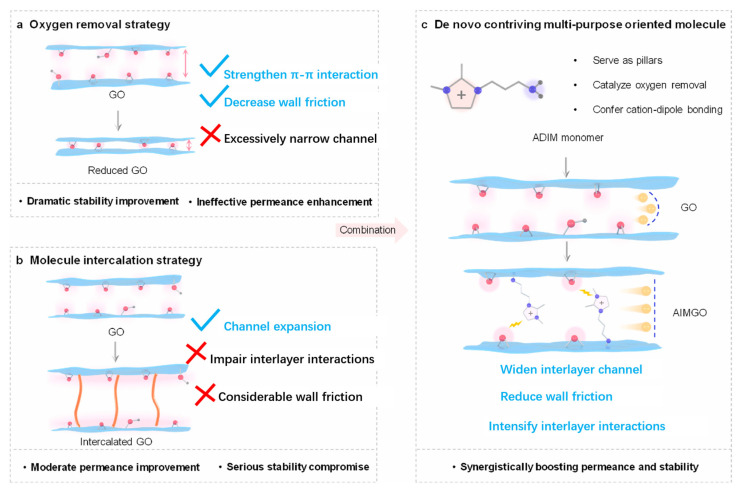
Schematic illustration of the de novo, three-in-one strategy for contriving an oriented molecule and its advantages [138]. Copyright 2024, Elsevier.

Apart from the exploration of fabrication methods, the interlayer spacing of GO plays a crucial role in determining both the water flux and the selectivity of graphene oxide membranes for OMPs. In the dry environment, the interlayer free spacing of GO is approximately 0.30–0.35 nm [134]. However, this spacing varies with ambient humidity, and the weak stability between the layers makes it difficult to achieve precise molecular separation [135]. Current research on GO-based NF membranes for separation of OMPs primarily focuses on balancing permeability and selectivity, as well as enhancing membrane resistance to fouling. To address these challenges, various modification strategies have been developed to adjust and optimize the physicochemical properties of GO membranes. Kong et al. found that changes in the interlayer spacing of GO have a direct impact on the rejection of organic pollutants [136]. GO-based membranes were fabricated by immobilizing GO nanosheets onto a polyvinylidene fluoride (PVDF) substrate using polydopamine (PDA) and tuning the interlayer spacing and membrane performance through functionalization with ethylenediamine (EDA) or β-cyclodextrin-functionalized EDA (β-CD-EDA). The rejection performance and mechanisms for three pharmaceuticals (propranolol, carbamazepine, sulfadiazine) with similar molecular weights (ranging from 236.27 to 259.34 Da) were investigated. The interlayer spacings of the EDA-crosslinked membrane (PDA-GO/EDA) and the β-CD-EDA-crosslinked membrane (PDA-GO/β-CD-EDA) were 1.13 nm and 0.97 nm, respectively, both significantly larger than that of the PDA-coated GO membrane (PDA-GO), which measured 0.78 nm. However, the stable rejection rates of the three drugs remained unsatisfactory, indicating the need for further performance enhancement through optimization of membrane fabrication and precise control over pore size distribution. In a follow-up study, the team improved the fabrication method by directly treating GO with dopamine, which increased the interlayer spacing from 0.78 nm to 1.02 nm and enhanced the water flux of the resulting GO/DA membrane [137]. By controlling the amount of DA added, they successfully prepared crosslinked GO membranes with both improved water permeability and excellent long-term stability in the separation of OMPs. However, the expansion of interlayer spacing inevitably weakens the interlayer interactions between adjacent nanosheets, leading to reduced long-term structural stability. To address this issue, Tan et al. designed a multifunctional, directionally grafted molecule, 1-(3-aminopropyl)-2,3-dimethylimidazolium bromide (ADIM) [138]. The imidazolium cationic groups interact with the oxygen-containing functional groups of GO, thereby enhancing the membrane’s resistance to swelling. As shown in Figure 9, the entire molecule functions like a pillar, maintaining the interlayer spacing. In this compound, the amino groups reduce GO and simultaneously act as covalent anchoring points, which helps enlarge the graphene regions and reduce resistance to molecular transport. The modified GO membrane (AIMGO) exhibited an order-of-magnitude increase in permeability compared to unmodified GO membranes, and achieved a separation factor of 9.8 for 4-dimethylaminopyridine, significantly surpassing that of state-of-the-art polymer membranes currently used for pharmaceutical purification.

#### 4.2.2. MXene/MoS_2_

MXenes are a class of two-dimensional nanomaterials composed of transition metal carbides, nitrides, or carbonitrides, with a general formula of M_n+1_X_n_T_x_, where M represents a transition metal (e.g., Ti, V, Nb), X denotes carbon (C) and/or nitrogen (N), and T_x_ stands for surface terminal groups such as –OH, –O, and –F [139]. As a two-dimensional GO-like material, MXenes are also suitable for fabricating NF membranes via vacuum/pressure-driven filtration. The edges, defects, and surfaces of MXene nanosheets are rich in functional groups, which contribute to enhanced hydrophilicity, functionalization potential, and separation selectivity. Arshad et al. prepared MXene-based MMMs and found that, despite using a smaller amount of material, the membrane achieved the highest caffeine removal efficiency among the control samples [140]. This could be attributed to the presence of defects in MXene, which introduced additional reactive sites and enhanced the catalytic performance of MnO_2_. Furthermore, the layered and highly hydrophilic structure of MXene improved adsorption capacity and promoted the generation of reactive oxygen species (ROS), thereby facilitating the effective degradation of caffeine. As a result, MXene proved to be highly beneficial in both the catalytic process involving MnO_2_ and the adsorption of caffeine. Ultimately, the caffeine molecules were broken down into smaller, less harmful compounds and completely mineralized.

Studies on two-dimensional GO-based materials have demonstrated that the layered structure of MXene serves as a mass transport channel, offering a promising strategy to overcome the permeability/selectivity trade-off commonly observed in polymer-based membranes. Nanosheets with a high aspect ratio tend to form more orderly interlayer structures, resulting in a narrower pore size distribution and consequently higher rejection rates of OMPs [141,142]. For example, Li et al. fabricated layered titanium carbide (Ti_3_C_2_T_x_) membranes with well-defined slit-like nanochannels by assembling ultra-large MXene nanosheets (2–4 mm in lateral size), and applied them for the separation of antibiotics from water [143]. These Ti_3_C_2_T_x_ membranes exhibited solvent permeabilities that were an order of magnitude higher than most polymer-based NF membranes, while maintaining a comparable antibiotic rejection performance. This high flux was attributed to the highly ordered two-dimensional (2D) structure formed by the large aspect ratio of the MXene nanosheets. A similar example was reported by Yue et al., who fabricated MXene-based hybrid nanosheets with large lateral dimensions (5–8 μm) [144]. By a vacuum-driven filtration method, the nanosheets were stacked onto a substrate to form NF membranes with well-aligned parallel slit-like channels and an interlayer spacing of 1.36 nm, which were applied for the purification of antibiotic-containing water. Compared to most polymeric and other two-dimensional NF membranes with similar rejection, these membranes exhibited a 100-fold increase in water flux.

MoS_2_ is a typical layered transition metal dichalcogenide with a sandwich-like S–Mo–S structure, where individual layers are stacked via weak van der Waals interactions. This unique structure endows MoS_2_ with excellent exfoliability and the characteristics of a 2D material. Although MoS_2_ has shown promising applications in fields such as electronics and catalysis, its limited surface functional groups, the difficulty in tuning interlayer spacing, and poor film-forming ability—compared to other 2D materials such as GO—have significantly constrained its development in NF membrane fabrication [123]. As a result, studies on MoS_2_-based NF membranes remain relatively scarce, and related applications are still in the exploratory stage. Dai et al. fabricated TFN membranes by incorporating MoS_2_ nanosheets into the PA selective layer. The embedded MoS_2_ nanosheets introduced hydrophilic surfaces and nanochannels within the PA layer by the IP process, aiming to enhance the removal of hydrophobic EDCs (methylparaben, ethylparaben, propylparaben, and benzylparaben) from wastewater. The results demonstrated that the incorporation of MoS_2_ nanosheets significantly improved both water flux and EDC rejection, with the water/EDC selectivity increasing by nearly sixfold compared to the control membrane. This enhancement was attributed to the suppressed hydrophobic interactions between the membrane surface and EDC molecules, as well as the selective transport properties induced by the MoS_2_-derived nanochannels.

### 4.3. Hybrid Materials and Environmentally Friendly Materials

Hybrid materials have emerged as promising candidates for NF membranes targeting OMPs, as they integrate the advantages of multiple components to enhance separation performance. For instance, a hybrid composed of GO nanosheets and muscovite nanosheets has been reported: multilayered exfoliated black mica (EB) nanosheets were prepared via liquid-phase exfoliation of natural EB and subsequently mixed with GO nanosheets at varying ratios [145]. This hybrid material was incorporated into polyethersulfone (PES) membranes, leading to a significant improvement in membrane performance. Compared to membranes without GO incorporation, the hybrid membranes exhibited markedly increased water flux and achieved a levofloxacin rejection rate of 80.3%. In order to achieve broad-spectrum rejection of various hydrophilic (both charged and uncharged, soluble and insoluble) and hydrophobic PhACs and PCPs, Oikawa et al. designed a novel membrane by embedding carbon quantum dot (CQD)-like NPs into GO membranes [146]. This unique design strategy enabled the modulation of interlayer charge environments within the GOM, allowing the coexistence of both positively and negatively charged functional groups within the slightly expanded, hydrophilic interlayer spacing. As a result, the membrane exhibited an enhanced separation performance toward a wide range of PCPs. A similar example of GO-based hybrid materials is the UiO-66/PGP TFC membrane developed by Fang et al., which integrates GO and MOFs as composite materials for an enhanced NF performance [147].

It is worth noting that certain macromolecular materials with unique cage-like structures have emerged as promising candidates for the fabrication of NF membranes targeting OMPs. These molecular cages are well-suited for blending with conventional polymer matrices due to their abundance of functional groups, which promote the formation of highly crosslinked networks and enhance pore uniformity across the membrane. Moreover, the tunable permeation pathways of these cage structures enable the development of multifunctional membranes. For instance, Li et al. fabricated TFN membranes via IP using porous organic cages (POCs), such as Tren and RCC3, in which highly catalytic Pd nanoclusters were embedded within the cages [148]. The resulting membrane exhibited not only a high separation efficiency for OMPs but also a self-cleaning function through catalytic degradation of adsorbed pollutants, demonstrating excellent multifunctionality. In addition to macromolecular materials, certain functionalized inorganic fillers have also been employed to fabricate NF membranes via in situ growth, demonstrating excellent performance in the removal of OMPs. The enhanced rejection of OMPs can be attributed to the improved membrane surface charge conferred by the functional fillers, while the increased water flux results from the formation of additional water transport pathways introduced by the fillers. For example, Jillani et al. fabricated an amine-functionalized zeolite-based NF membrane via an in situ growth method [148]. Using caffeine, sulfamethoxazole, amitriptyline, and loperamide as representative OMPs, the membrane achieved rejection rates exceeding 95% for all target compounds. Moreover, numerous emerging materials, such as 2D nanomaterials, POCs, and biomass-derived components, hold great promise for application in OMP removal, presenting a rich landscape for future exploration and innovation [149,150].

The application of renewable materials in the fabrication of nanofiltration membranes should be emphasized to enhance the environmental sustainability of membrane separation technologies. Furthermore, novel environmentally friendly materials such as cellulose and chitosan also show great potential in this field. Zhang et al. developed a low-cost strategy for fabricating TpPa-based wood-derived NF membranes by in situ growth of imine-linked COFs on the top surface of pretreated wood blocks [151]. By utilizing renewable wood as the membrane substrate, the abundant hydroxyl groups in cellulose provided numerous nucleation sites for the formation of dense and defect-free COFs with an average pore size of approximately 1.8 nm. In addition, the TpPa–Wood membrane leverages the advantages of biomass substrates, featuring abundant microchannels and high hydrophilicity, which significantly enhanced the mechanical robustness and separation performance of the membrane for effective removal of OMPs (norfloxacin and tetracycline). Asad et al. fabricated chitosan-based porous membranes, fully utilizing the material’s renewability and excellent film-forming properties, and highlighted the potential of chitosan as an adsorptive support material in membrane separation applications [140].

**Figure 10:**
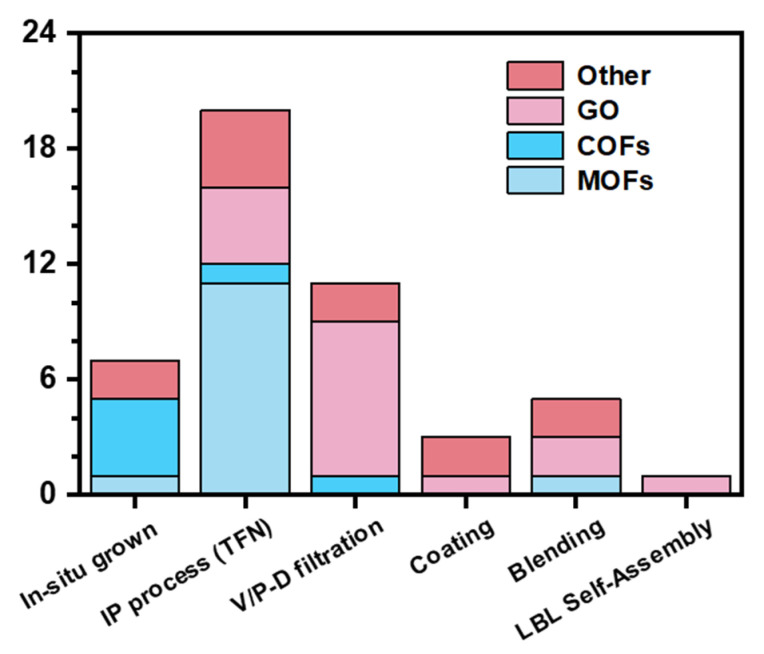
Comparative analysis of material contributions in NF membrane fabrication methods.

To give a brief summary, we have systematically reviewed the materials, separation operations, and separation performance for OMPs in NF membranes in recent years (as summarized in Table 5). Among these, organic framework materials, particularly MOFs, COFs, and GO, have been the focus of the most intensive and extensive exploration in the fabrication of NF membranes for OMP removal. In terms of operational modes, cross-flow filtration has been widely adopted by researchers due to its superior antifouling properties compared to dead-end filtration. The selected operational pressures for membrane testing range from 0.53 to 25 bar, with most studies focusing around 5 bar. Inorganic layered materials such as GO and MXene exhibit higher tolerance to separation pressure compared to organic framework materials, primarily because they are generally assembled into NF membranes via V/P-D filtration, resulting in more robust mechanical integrity. Conversely, organic framework materials are typically fabricated through in situ IP or integrated into PA layers via the IP process, during which defects may form that limit the membranes’ mechanical strength under high pressure (Figure 10). However, 2D organic frameworks possess both a layered structure and framework characteristics, suggesting that after filtration-assisted deposition, 2D COFs could potentially yield NF membranes with enhanced mechanical properties. The reported concentration range for OMP separation spans from 0.00125 to 250 ppm. Among the cases with water permeance exceeding 50 LMH/bar, 40% utilized COF-based membranes, and 80% were related to organic framework materials, underscoring the crucial role of their porosity and functional groups in enhancing water flux. Nevertheless, due to the diversity in OMP species, concentrations, and operating pressures selected across different studies, rigorous cross-study performance comparisons remain challenging. Overall, these advanced organic framework materials, inorganic layered materials, hybrid materials, and other novel materials provide abundant opportunities for the design and fabrication of high-efficiency OMP-separating NF membranes (as summarized in Table 6), demonstrating significant potential for industrial applications.

## 5. Challenges and Future Perspectives

### 5.1. Scalable Fabrication Process

Beyond membrane separation itself, the development of reproducible and scalable manufacturing processes for membranes is a core challenge for future research. From the perspective of industrial-scale production, current fabrication technologies for NF membranes aimed at the removal of OMPs, including the IP process, MMM fabrication, coating, and V/P-D filtration deposition (particularly for hollow fiber membranes), demonstrate strong potential for commercialization. For organic framework materials, the most convenient approach for large-scale membrane fabrication is typically through blending to form a PA selective layer. In the case of 2D layered inorganic materials, large-scale membrane production is commonly achieved via deposition onto hollow fiber membranes. However, it is important to consider that the cross-section of hollow fiber membranes presents a curved, cylindrical surface as the substrate, which may influence coating uniformity and interfacial compatibility. Consequently, research into scalable fabrication strategies for membranes based on these new materials remains limited. Moving forward, the industrial-scale production of OMP-removing NF membranes necessitates the development of customized fabrication processes tailored to the unique properties of each novel material.

### 5.2. Environmental Sustainability Goals

While advanced materials offer remarkable advantages in enhancing nanofiltration performance for OMP removal, their environmental sustainability remains a significant challenge. Currently, many membrane materials are derived from petroleum-based polymers, which are non-biodegradable and pose disposal issues after use. Therefore, the development and application of renewable and biodegradable materials (such as chitosan, cellulose, and lignin) should be prioritized to align with environmental sustainability goals. Moreover, the disposal of spent membranes is an emerging concern. With the increasing deployment of membrane technologies in wastewater treatment, membrane waste is also growing. Common disposal methods such as landfilling and incineration may cause secondary pollution. Advancing membrane recycling technologies or using naturally degradable materials could alleviate environmental burdens.

### 5.3. Complex Separation System

In the field of OMP separation, the vast majority of studies to date have primarily focused on exploring new materials with singular separation characteristics and calculating their selectivity. While these measurements are highly precise, they should not be regarded as the optimal approach for characterizing new materials in realistic OMP separation scenarios. In complex aqueous systems containing OMPs, the separation processes are often influenced by interactions between different OMPs, some of which are synergistic while others are antagonistic. Therefore, future research should place a greater emphasis on investigating separation in complex water matrices, including studies using real-world water systems [164].

## 6. Conclusions

This review first provides an overview of the sources and release pathways of organic micropollutants (OMPs), including pharmaceutically active compounds (PhACs), endocrine-disrupting compounds (EDCs), and personal care products (PCPs). It then examines their environmental pollution, including water and plant contamination, bioaccumulation in animals, and potential human health risks. In addressing the challenges posed by OMPs in the environment, this review focuses particularly on membrane separation technologies for OMP removal from water and wastewater, in addition to conventional treatments and other advanced technologies. It emphasizes the role of NF (NF) membranes, examining how key properties, including pore size, surface charge, interaction sites, and hydrophilicity, affect membrane performance, and the strategies employed to modulate these characteristics. In the final section, this review details recent advancements over the past 15 years in novel materials used for OMP separation via NF, including organic frameworks (e.g., MOFs, COFs, HOFs, and POFs), inorganic layered materials (e.g., GO, MXene, and MoS_2_), and various hybrid materials. In conclusion, while significant progress has been made in developing novel NF materials for OMP removal, key challenges remain. These include the lack of robust mathematical models of separation mechanisms, limited studies linking mechanisms to performance, inadequate scalable fabrication methods for new materials, and insufficient research on OMP separation in complex water matrices. Future efforts should focus on developing predictive models, scalable fabrication tailored to novel materials, and enhancing membrane performance for multicomponent separation, real wastewater treatment, and continuous operations.

## Figures and Tables

**Table 1:** OMP classification based on the reviewed literature.

OMPs	Sub-Class	Chemicals
PhACs	Antibiotics	Amoxicillin, Cephalexin, Rifampicin, Bacitracin, Azithromycin, Erythromycin, Tetracycline, Penicillin, Ampicillin Sodium, Thiamphenicol, Oxytetracycline, Chlortetracycline, Clarithromycin, Roxithromycin, Tylosin, Ciprofloxacin, Norfloxacin, Levofloxacin, Lincomycin
	Analgesics/ Antipyretics/ Anti-inflammatory	Paracetamol, Acetaminophen, Ibuprofen, Naproxen, Diclofenac, Mefenamic acid, Aspirin, Ketoprofen, Indometacin, Clofibric acid, Bezafibrate, Fenoprofen, Ethenzamide, Antipyrine, Isopropylantipyrine
	Insecticides	DEET, Crotamiton, Atrazine, Propazine, Prometryn
	Antiepileptic	Carbamazepine, Primidone
	β-receptor blockers/Agonist	Atenolol, Propranolol, Salbutamol, Metoprolol, Terbutaline
	Antipsychotic/Antidepressants	Fluoxetine, Sulpiride, Amitriptyline, Sertraline, Paroxetine, Nortriptyline, Doxorubicin, Omeprazole, Diltiazem
PCPs	Preservatives (Parabens)	Methylparaben, Propylparaben, Ethylparaben, Benzylparaben
	Antimicrobials/Disinfectants	Triclosan, Triclocarban
	Surfactants/Dyes/Miscellaneous	Congo red, Methyl blue, Rhodamine B, Calcein, Coomassie brilliant blue, Sodium 2-biphenylate, DEET
EDCs	Hormones	17α-Ethynylestradiol
	Hormone-like/Metabolic regulator	Bisphenol A, Bisphenol AF, Sodium 2-biphenylate, Metformin, Gemfibrozil
Others	Loperamide HCl, Caffeine, Omeprazole, Dipyridamole, Iodixanol, Berberine chloride	

**Table 2:** E-factors, waste, and process complexity across different industries. (The statistics are based on the data from 2020 to 2025.)

Industry Segment (e.g.)	Annual Product Tonnage (Each)	E-Factor (kg Waste/ kg Product)	Total Annual Waste Tonnage	Synthetic Steps	Years of Development
Petrochemicals (solvents, detergents) [25]	5 × 10^9^	0.1–5	10^9^	-	110+
Bulk chemicals (plastics, polymers) [26]	5 × 10^8^	1–5	2 × 10^9^	1–2	20–60
Fine chemicals (coatings, electronic parts, pharmaceutical raw materials) [27]	10^6^–10^7^	5–50	50 × 10^8^	3–4	14–17
Pharmaceuticals (antibiotics, drugs, vaccines) [28]	10^4^–10^5^	50–100	10^7^	5+	13–15

**Table 3:** Information on the molecular formula, molecular weight, charge characteristics, and van der Waals volume of OMPs.

OMP Name	Molar Mass (Da)	Formula	Charge (pH = 7)	Van der Waals Volume (Å3)
Methylparaben	152.1	C_8_H_8_O_3_	Neutral	135.76
Paracetamol	151.2	C_8_H_9_NO_2_	Neutral	138.08
Ethylparaben	166.2	C_9_H_10_O_3_	Neutral	152.62
Ethenzamide	165.2	C_9_H_11_NO_2_	Neutral	155.37
Caffeine	194.2	C_8_H_10_N_4_O_2_	Neutral	164.26
Propylparaben	180.2	C_10_H_12_O_3_	Neutral	169.57
Antipyrine	188.2	C_11_H_12_N_2_O	Neutral	174.44
Atrazine	215.7	C_8_H_14_ClN_5_	Neutral	190.9
DEET	191.3	C_12_H_17_NO	Neutral	198.31
Primidone	218.3	C_12_H_14_N_2_O_2_	Neutral	200.65
Benzylparaben	228.2	C_14_H_12_O_3_	Neutral	206.36
Crotamiton	203.3	C_13_H_17_NO	Neutral	207.44
Propazine	229.7	C_9_H_16_ClN_5_	Neutral	207.98
Carbamazepine	236.3	C_15_H_12_N_2_O	Neutral	210.15
Triclosan	289.5	C_12_H_7_Cl_3_O_2_	Neutral	212.06
Bisphenol A	228.3	C_15_H_16_O_2_	Neutral	221.5
Isopropylantipyrine	230.3	C_14_H_18_N_2_O	Neutral	225.4
Prometryn	241.3	C_10_H_19_N_5_S	Neutral	229.85
Triclocarban	315.6	C_13_H_9_Cl_3_N_2_O	Neutral	236.67
Bisphenol AF	336.4	C_15_H_10_F_6_O_2_	Neutral	251.77
Ampicillin Sodium	371.4	C_16_H_18_N_3_NaO_4_S	Neutral	269.23
Norfloxacin	319.3	C_16_H_18_FN_3_O_3_	Neutral	277.46
Thiamphenicol	356.21	C_12_H_15_C_l2_NO_5_S	Neutral	278.94
Ciprofloxacin	331.3	C_17_H_18_FN_3_O_3_	Neutral	282.83
17α-Ethynylestradiol	296.4	C_20_H_24_O_2_	Neutral	291.82
Omeprazole	345.4	C_17_H_19_N_3_O_3_S	Neutral	301.14
Levofloxacin	361.4	C_18_H_20_FN_3_O_4_	Neutral	309.96
Rhodamine B	479	C_28_H_31_ClN_2_O_3_	Neutral	422.26
Rifampicin	822.9	C_43_H_58_N_4_O_12_	Neutral	759.07
Iodixanol	1550.2	C_35_H_44_I_6_N_6_O_15_	Neutral	852.98
Bacitracin	1422.7	C_66_H_103_N_17_O_16_S	Neutral	1314.36
Metformin	129.2	C_4_H_11_N_5_	Positive	123.58
Aspirin	180.2	C_9_H_8_O_4_	Positive	154.85
Clofibric acid	214.7	C_10_H_11_ClO_3_	Positive	184.05
Ibuprofen	206.3	C_13_H_18_O_2_	Positive	211.8
Naproxen	230.3	C_14_H_14_O_3_	Positive	213.06
Terbutaline	225.3	C_12_H_19_NO_3_	Positive	222.28
Fenoprofen	242.3	C_15_H_14_O_3_	Positive	223.44
Mefenamic acid	241.3	C_15_H_15_NO_2_	Positive	225.99
Ketoprofen	254.3	C_16_H_14_O_3_	Positive	233.68
Diclofenac	296.1	C_14_H_11_Cl_2_NO_2_	Positive	236.85
Salbutamol	239.3	C_13_H_21_NO_3_	Positive	239.15
Propranolol	259.3	C_16_H_21_NO_2_	Positive	257.56
Atenolol	266.3	C_14_H_22_N_2_O_3_	Positive	261.34
Nortriptyline	263.4	C_19_H_21_N	Positive	265.01
Sertraline	306.2	C_17_H_17_C_l2_N	Positive	266.82
Fluoxetine	309.3	C_17_H_18_F_3_NO	Positive	274.24
Metoprolol	267.4	C_15_H_25_NO_3_	Positive	274.25
Amitriptyline	277.4	C_20_H_23_N	Positive	282.76
Berberine chloride	371.8	C_20_H_18_NO_4_Cl	Positive	292.73
Paroxetine	329.4	C_19_H_20_FNO_3_	Positive	293.59
Indometacin	357.8	C_19_H_16_ClNO_4_	Positive	300.84
Sulpiride	341.4	C_15_H_23_N_3_O_4_S	Positive	307.33
Bezafibrate	361.8	C_19_H_20_ClNO_4_	Positive	319.67
Diltiazem	414.5	C_22_H_26_N_2_O_4_S	Positive	378.6
Lincomycin	406.5	C_18_H_34_N_2_O_6_S	Positive	384.58
Loperamide HCl	477	C_29_H_33_ClN_2_O_2_	Positive	451.19
Doxorubicin	543.5	C_27_H_29_NO_11_	Positive	463.44
Dipyridamole	504.6	C_24_H_40_N_8_O_4_	Positive	476.19
Erythromycin	733.9	C_37_H_67_NO_13_	Positive	727.13
Clarithromycin	747	C_38_H_69_NO_13_	Positive	744.46
Azithromycin	749	C_38_H_72_N_2_O_12_	Positive	754.48
Roxithromycin	837	C_41_H_76_N_2_O_15_	Positive	824.52
Tylosin	916.2	C_46_H_77_NO_17_	Positive	882.63
Penicillin	243	C_9_H_11_N_2_O_4_	Negative	225.18
Gemfibrozil	250.3	C_15_H_22_O_3_	Negative	255.17
Cephalexin	347.4	C_16_H_17_N_3_O_4_S	Negative	290.71
Amoxicillin	365.4	C_16_H_19_N_3_O_5_S	Negative	307.12
Tetracycline	444.4	C_22_H_24_N_2_O_8_	Negative	379.67
Oxytetracycline	460.4	C_22_H_24_N_2_O_9_	Negative	388.09
Chlortetracycline	478.9	C_22_H_23_ClN_2_O_8_	Negative	396.69
Penicillin	243	C_9_H_11_N_2_O_4_	Negative	225.18

Data from Chemicalize library, https://chemicalize.com (accessed on 30 May 2025).

**Table 4:** Classification of organic frameworks and their characteristics.

Types of Organic Framework	Nodes	Chemical Bonds	Crystallinity
MOFs	Metal ions or clusters	Coordination bond	High
POFs	Organic ligand	Chemical bond	Low, Moderate
COFs	Organic ligand	Covalent bond	Moderate or high
HOFs	Organic ligand	Hydrogen bond	Moderate or high

**Table 5:** Membrane name, separation mode, and performance summary.

Membrane	Mode	Press. (bar)	Separation Performance (OMPs; Concentration, ppm; Flux, LMH/bar; Rejection, %)	Refs.
MIL-101(Fe)-NH2-loaded MAq	cross	5	Fluoxetine hydrochloride; 50; 7.76; 81.9 Sertraline hydrochloride; 50; 7.76; 75.8 Paroxetine hydrochloride; 50; 7.76; 71.5 Nortriptyline hydrochloride; 50; 7.76; 73.1	[109]
PES/MOF-808@PA TFN	dead-end	4	Aspirin; 10; 6.04; 88.76 Naproxen; 10; 6.04; 84.51 Mefenamic acid; 10; 6.04; 70.56	[110]
MOF/PA-TFC	cross	5	Paracetamol; 50; ~3.5; 93 Ibuprofen; 50; ~3.5; 98 Amoxicillin; 50; ~3.5; 99	[113]
SurZn3/SubCo3	cross	4	Doxorubicin; 25; 4.41; 89.0	[112]
AMSA-MIL-101(Cr)	cross	8	Terbutaline; 0.2; 23; ~83 Atenolol; 0.2; 23; ~90 Fluoxetine; 0.2; 23; ~93 Ketoprofen; 0.2; 23; ~90 Diclofenac; 0.2; 23; ~90 Bezafibrate; 0.2; 23; ~90	[111]
PSF-ZIF-8/PA	dead-end	4	Paracetamol; 100; 3.5; ~55	[152]
TFN NH2-UiO-66-PL-7.6	cross	10	Phenacetine; 0.5; 6.97; ~67 Nalidixic acid; 0.5; 6.97; ~85 Carbamazepine; 0.5; 6.97; ~76 Sulfamethoxazole; 0.5; 6.97; ~82 Atenolol; 0.5; 6.97; ~78 Sulpiride; 0.5; 6.97; ~92	[153]
PA ZIF-93 BTFC PA HKUST-1 BTFC	dead-end	20	Diclofenac; 1; 33.1; 99.5 Naproxen; 1; 24.9; 99	[154]
Ag@UiO-66-NH2/PA	cross	8	Biphenol A; 50; 8.12; 94.6	[155]
TAS-Z-PiP-TFN	cross	13.8	Sulfamethoxazole; 10; 3.6; ~30 Amitriptyline; 10; 3.6; ~64 Omeprazole; 10; 3.6; ~60 Loperamide HCl; 10; 3.6; ~70	[156]
MOF0.20-TFN	cross	8	Methylparaben; 0.2; 39.5; 47.4 Propylparaben; 0.2; 39.5; 45.9 Benzylparaben; 0.2; 39.5; 51.1 Bisphenol A; 0.2; 39.5; 79.8	[157]
TFN-COF0.05	cross	4	Bisphenol A; 2; 17.1; 98.3 Bisphenol AF; 5; 17.1; 99.1 Sodium 2-biphenylate; 5; 17.1; 99.3	[115]
COF TpPa-SO3H	cross	5	Diclofenac; 200; 1.67; 96.4 Ketoprofen; 20; 1.67; 75.8 Naproxen; 15; 1.67; 75.2 Ibuprofen; 20; 1.67; 79.4 Sulfamethoxazole; 300; 1.67; 57.4	[114]
TpPa-PEI 0.125%-10	cross	5	Sulfamethazine; 0.2; 4.0; 62.1 Carbamazepine; 0.2; 4.0; 58.7 Propranolol; 0.2; 4.0; 94.1 Sulpiride; 0.2; 4.0; 97.2 Dametformin; 0.2; 4.0; 71.2	[116]
TpTAPA/HPAN	cross	3	Ammonium glycyrrhizinate; 100; 68.1; 92 Diammonium glycyrrhizinate; 100; 68.1; 92.0	[117]
COF-LZU1	cross	5	Tetracycline; 0.4; 23.3; 82.5 Sulfadiazine; 0.4; 23.3; 78.0 Carbamazepine; 0.4; 23.3; 78.4 Propranolol; 0.4; 23.3; 73.6	[118]
HOF-TFN-2	cross	1	Congo red; 0.25 mmol/L; 546; 95.85 Coomassie brilliant blue; 0.25 mmol/L; 546; 96.47 Hodamine B; 0.25 mmol/L; 546; 97.26 Rmethyl blue; 0.25 mmol/L; 546; 83.70 Calcein; 0.25 mmol/L; 546; 92.59	[95]
BILP-101x/HPAN	cross	4	Congo red; 0.2; 235; 99 Methyl blue; 0.2; 235; 92 Direct red 23; 0.2; 235; 99 Rhodamine B; 0.2; 235; 90	[119]
GO-coated ceramic hollow fiber	cross	5	Rifampicin; 20; 3.5; 52 Propranolol; 0.5; 2.4; 32	[129]
TFN1	cross	5	Sulfamethoxazole; 100; 4.46; 96 Triclosan; 100; 4.46; 94 Diclofenac; 100; 4.46; 91 Cephalexin; 100; 4.46; 92	[132]
GO NF/RGO NF	cross	20	Ibuprofen; 10; 94; 89	[128]
TFC-GO	cross	6.8	Sulfamethazone; 0.8; 0.44; ~55 Ibuprofen; 0.8; 0.44; ~45 Triclosan; 0.8; 0.44; ~62 Sulfadiazine; 0.8; 0.44; ~58 Sulfamethoxazole; 0.8; 0.44; ~56 Carbamazepine; 0.8; 0.44; ~57	[133]
PDA-GO/β-CD-EDA	dead-end	5	Carbamazepine; 0.5;6.8; ~16 Sulfadiazine; 0.5; 6.8; ~55 Propranolol; 0.5; 6.8; ~63	[136]
GO membrane	cross	5	Secondary effluent; 175 mgO2/L; 10; 76	[126]
30 nm GO membrane	dead-end	2	Gemfibrozil; 10; -; 76.4 17α-ethynylestradiol; 10; -; 80.1 Diclofenac sodium salt; 10; -; 83 Iodixanol; 10; -; 95.2	[130]
Ceramic GO membrane	dead-end	3	Ibuprofen; 10; ~5; 58 Sulfamethoxazole; 10; ~5; 48	[127]
hPAN + GO	dead-end	20.7	Triclosan; 0.00125; ~2.5; 95 Triclocarban; 0.00125; ~2.5; 99	[131]
GO/25%DA	dead-end	5	Propranolol; 0.2; ~4.6; 40 Carbamazepine; 0.2; ~4.6; 38 Sulfadiazine; 0.2; ~5.0; 50	[137]
AIMGO-3 membrane	dead-end	1	Roxithromycin; 10; -; 90.2 4-dimethylaminopyridine; 1; -; 92.0	[138]
GO-modified membranes	cross	15	Amitriptylene HCl; 10; ~2; ~90 Bisphenol-A; 10; ~2; ~100 Acetaminophen; 10; ~2; ~90 Caffeine; 10; ~2; ~80	[158]
PA/GO-4	cross	6	Paracetamol; 1; ~10; 4.63 Norfloxacin; 1; ~10; 53.32 Sulfamethoxazole; 1; ~10; 41.85	[159]
MXMn membrane	dead-end	7	Caffeine; 1; 19.33; 99.99	[140]
Ti3C2Tx membranes	dead-end	1	Bacitracin; 250; ~370; ~94 Azithromycin; 250; ~350; ~85 Erythromycin; 250; ~350; ~85 Tetracycline; 250; ~325; ~80 Penicillin; 250; ~305; ~78	[143]
MP30 membrane	dead-end	1	Ampicillin sodium; 200; 287.5; ~90.4 Berberine chloride; 200; 291.2; ~92.9 Tetracycline; 200; 300.8; ~92.5 Erythromycin; 200; 318.8; ~94.6	[144]
MNF2	cross	10	Methylparaben; 0.2; -; 53.7 Ethylparaben; 0.2; -; 69.1 Propylparaben; 0.2; -; 79.1 Benzylparaben; 0.2; -; 91.3	[160]
B:G (1:1) membrane	dead-end	4	Levofloxacin; 100; ~11; ~85	[145]
TpPa-wood membrane	dead-end	0.53	Norfloxacin NFX; 20; 1200; ~96 Tetracycline TC; 20; 1200; ~94	[151]
EDA-CQD_GOM	cross	~	Clofibric acid; -; -; ~90 Naproxen; -; -; ~90 Mefenamic acid; -; -; ~99 Fenoprofen; -; -; ~90 Ketoprofen; -; -; ~92 Diclofenac; -; -; ~93 Furosemide; -; -; ~88 Indometacin; -; -; ~98 Bezafibrate; -; -; ~90 Acetaminophen; -; -; ~60 Ethenzamide; -; -; ~78 Theophylline; -; -; ~62 Antipyrine; -; -; ~80 DEET; -; -; ~84 Caffeine; -; -; ~70 Crotamiton; -; -; ~80 Primidone; -; -; ~62 Isopropylantipyrine; -; -; ~78 Sulfathiazole; -; -; ~60 Cyclophosphamide; -; -; ~70 Sulfamerazine; -; -; ~69 Sulfadimidine; -; -; ~69 Sulfamonomethoxine; -; -; ~83 Thiamphenicol; -; -; ~81 Oxytetracycline; -; -; ~88 Chlortetracycline; -; -; ~99 Dipyridamole; -; -; ~96 Salbutamol; -; -; ~61 Atenolol; -; -; ~60 Trimethoprim; -; -; ~60 Sulpiride; -; -; ~60 Lincomycin; -; -; ~63 Diltiazem; -; -; ~82 Tiamulin; -; -; ~98 Clarithromycin; -; -; ~70 Roxithromycin; -; -; ~75 Tylosin; -; -; ~82	[146]
UiO-66/PGP TFC	cross	3	Oxytetracycline HCI; 10; 17.61; 94.8 Tetracycline hydrochloride; 10; 17.61; 95.5 Ciprofloxacin; 10; 16.09; 98.6 Sulfamethoxazole; 10; 27.46; 83.05	[147]
poly-Pd@RCC3/PAN	dead-end	1	Chlorophyll; 20; 7.5; ~97 Flavonoids; 20; 7.5; ~47 Ellagitannins; 20; 7.5; ~45	[89]
Pr-MCM-41-NH2-PA/PSf	cross	25	Caffeine; -; ~2.24; ~97 Sulfamethoxazole; -; ~2.24; ~97 Amitriptyline; -; ~2.24; ~97 Loperamide; -; ~2.24; ~97	[148]
ZnO membrane	cross	5	Atenolol; 0.09; 567; 96 Ibuprofen; 0.582; 561; 99	[150]
MTC	dead-end	7	Paracetamol; 5; 19.33; 90.98 Ibuprofen; 5; 19.33; 90.98	[149]
PA/TNT TFC	dead-end	15	Bisphenol A; 10; ~1; 89.05 Caffeine; 10; ~1; 97.89	[161]
S_2_	cross	10	Atrazine; 0.1; 2.9; ~97 Propazine; 0.1; 1.2; ~91 Prometryn; 0.1; 2.5; ~98	[162]
NF90-C0.5Ag4	cross	8	Ethylparaben; 200; 6.7; 67 Propylparaben; 200; 6.7; 69 Benzylparaben; 200; 6.7; 66 Bisphenol A; 200; 6.7; 99	[163]

**Table 6:** Representative membranes from literature: Composition, functionalization, and separation mechanisms.

Membrane	Material	Functional Group/Active Site	Separation Mechanism	Refs.
MIL-101(Fe)-NH_2_-loaded MAq	MIL-101(Fe)-NH_2_	-NH_2_	Electrostatic interaction, Hydrophilicity	[109]
PES/MOF-808@PA TFN	MOF-808	-COOH	Size exclusion, Electrostatic interaction	[110]
MOF/PA-TFC	Zn-MOF	-	Electrostatic interaction, Size exclusion	[113]
SurZn3/SubCo3	ZIF-8/ZIF-67	Metal oxide	Molecular interaction, Electrostatic interaction	[112]
AMSA-MIL-101(Cr)	MIL-101(Cr)	-NH_2_/-COOH	Electrostatic interaction	[111]
PSF-ZIF-8/PA	ZIF-8	~	Size exclusion	[152]
TFN NH2-UiO-66-PL-7.6	NH2-UiO-66	-NH_2_	Size exclusion, Electrostatic interaction	[153]
PA ZIF-93 BTFC PA HKUST-1 BTFC	ZIF-93/ HKUST-1	-OH	Size exclusion, Hydrophilicity	[154]
Ag@UiO-66-NH_2_/PA	UiO66-NH_2_	NH_2_/Ag NPs	Size exclusion, Electrostatic interaction	[155]
TAS-Z-PiP-TFN	ZnO	NH_2_	Size exclusion, Hydrophilicity	[156]
MOF0.20-TFN	MIL-101(Cr)	-	Hydrophilicity, Size exclusion	[157]
TFN-COF0.05	TPB-DMTP	-O-CH3	Hydrophilicity, Molecular interaction	[115]
COF TpPa-SO_3_H	TpPa-SO_3_H	-SO_3_H	Electrostatic interaction, Size exclusion	[114]
TpPa-PEI 0.125%-10	TPPA	-NH_2_	Electrostatic interaction, Hydrophilicity	[116]
TpTAPA/HPAN	TPTAPA	-OH	Size exclusion, Electrostatic repulsion	[117]
COF-LZU1	LZU-1	-OH	Electrostatic interaction, Size exclusion	[118]
HOF-TFN-2	Nano-PFC-1	-OH	Hydrophilicity, Size exclusion	[95]
BILP-101x/HPAN	BILP-101x	~NH~	Electrostatic interaction, Size exclusion	[119]
GO-coated ceramic hollow fiber	GO	OH	Size exclusion	[129]
TFN1	GO-NH_2_	-NH_2_	Size exclusion, Hydrophilicity	[132]
GO NF/RGO NF	GO/RGO	-OH/-COOH	Electrostatic interaction	[128]
TFC-GO	GO	-OH/-COOH	Electrostatic interaction	[133]
PDA-GO/EDA PDA-GO/β-CD-EDA	GO	-OH/-COOH	Size exclusion	[136]
GO membrane	GO	-OH/-COOH	Electrostatic interaction, Size exclusion	[126]
30 nm GO membrane	GO	-OH/-COOH	Size exclusion	[130]
Ceramic GO membrane	GO	-OH/-COOH	Size exclusion, Electrostatic interaction	[127]
hPAN + GO	GO	-OH/-COOH/-NH_2_	Size exclusion	[131]
GO/25%DA	GO	-OH/-COOH/-NH_2_	Size exclusion	[137]
AIMGO-3 membrane	GO	-OH/Imidazole cationic	Size exclusion	[138]
GO-modified membranes	GO	-NH_2_	Size exclusion, Electrostatic interaction	[158]
PA/GO-4	GO	-OH/-COOH	Size exclusion, Hydrophilicity, Electrostatic interaction	[159]
MXMn membrane	MXene	MnO_2_	Molecular interaction	[140]
Ti3C2Tx membranes	MXene	-	Size exclusion	[143]
MP30 membrane	MXene	-OH	Size exclusion, Electrostatic interaction	[144]
MNF2	MoS_2_	-	Size exclusion, Electrostatic interaction	[160]
B:G (1:1) membrane	GO + EB	OH	Size exclusion, Electrostatic interaction	[145]
TpPa-wood membrane	COF + wood	OH	Hydrophilicity, Size exclusion	[151]
EDA-CQD_GOM	GO+ CQD	OH/Pyridine	Size exclusion, Electrostatic interaction	[146]
UiO-66/PGP TFC	MOF + GO	-OH	Size exclusion, Electrostatic interaction	[147]
poly-Pd@RCC3/PAN	POC + Pd	Pd NCs	Size exclusion	[89]
Pr-MCM-41-NH_2_-PA/PSf	MCM-41	-NH_2_	Electrostatic interaction	[148]
ZnO membrane	ZnO	-	Hydrophilicity	[150]
MTC	Chitosan	MnO_2_	Hydrophilicity, Molecular interaction	[149]
PA/TNT TFC	TiO_2_	-OH	Size exclusion, Molecular interaction	[161]
S_2_	SiO_2_	Silane	Hydrophilicity, Electrostatic interaction	[162]
NF90-C0.5Ag4	Ag NPs	OH-NH_2_	Hydrophilicity, Size exclusion	[163]

## Data Availability

No new data were created or analyzed in this study. Data sharing is not applicable to this article.

## Abbreviations

The following abbreviations are used in this manuscript:

PhACs	pharmaceutically active compounds
PCPs	personal care products
EDCs	endocrine disrupting compounds
OMPs	organic micropollutants
NF	nanofiltration
MOFs	metal–organic frameworks
COFs	covalent organic frameworks
GO	graphene oxide
MWCO	molecular weight cut-off
AOPs	advanced oxidation processes
RO	reverse osmosis
FO	forward osmosis
ISA	integrally skinned asymmetric
TFC	membrane: thin-film composite membrane
TFN	membrane: thin-film nanocomposite membrane
NIPS	non-solvent-induced phase separation
LbL	layer by layer
PEI	polyethyleneimine
PVDF	poly (vinylidene fluoride)
BSA	bovine serum albumin
GA	gallic acid
PEG	polyethylene glycol
PSf	polysulfone
DABA	3,5-diaminobenzoic acid
HOFs	hydrogen-bonded organic frameworks
POFs	porous organic frameworks
PA	polyamide
ED	ethylenediamine
PDA	polydopamine
V/P-D	vacuum/pressure-driven
NPs	nanoparticles
IP	interface polymerization
Tp	1,3,5-triformylphloroglucinol
Pa	p-phenylenediamine
ICP	interfacial catalytic polymerization
PTSA	p-toluenesulfonic acid
EDA	ethylenediamine
β-CD-EDA	β-cyclodextrin-functionalized EDA
ROS	reactive oxygen species
CQD	carbon quantum dot
POCs	porous organic cages
PES	polyethersulfone
